# A Two-Step Variable Selection Strategy for Multiply Imputed Survival Data Using Penalized Cox Models

**DOI:** 10.3390/bioengineering12111278

**Published:** 2025-11-20

**Authors:** Qian Yang, Bin Luo, Chenxi Yu, Susan Halabi

**Affiliations:** 1 Division of Infectious Diseases, Department of Medicine, Emory University School of Medicine, Atlanta, GA 30322, USA; qian.yang@emory.edu; 2 School of Data Science and Analytics, Kennesaw State University, Marietta, GA 30060, USA; bluo@kennesaw.edu; 3 Department of Biostatistics and Bioinformatics, Duke University, Durham, NC 27708, USA; chenxi.yu@duke.edu

**Keywords:** multiple imputation, penalized method, proportional hazards model, missing data

## Abstract

Multiple imputation (MI) is widely used for handling missing data. However, applying penalized methods after MI can be challenging because variable selection may be inconsistent across imputations. We propose a two-step variable selection method for multiply imputed datasets with survival outcomes: apply LASSO or ALASSO to each MI dataset, followed by ridge regression, and combine estimates using variable selected in any or d% (d = 50, 70, 90, 100) of the MI datasets. For comparison, we also fit stacked MI datasets with weighted penalized regression and a group LASSO approach that enforces consistent selection across imputations. Simulations with Cox models evaluated tuning by AIC, BIC, cross-validation at the minimum error, and the 1SE rule. Across scenarios, performance differed by both the penalization and the selection rule. More conservative choices such as ALASSO with BIC and a 50% inclusion frequency tended to control false positive and gave more stable calibration. The grouped approach achieved comparable selection with modestly higher estimation error. Overall, no single method consistently outperformed others across all scenarios. Our findings suggest that practitioners should weigh trade-offs between selection stability, estimation accuracy, and calibration when applying penalized methods to multiply imputed survival data.

## 1. Introduction

Clinical studies are often hampered with missing data [1]. The missingness could be due to nonresponse, early dropout, or data collection errors. The nature of missingness is typically classified into three groups, based on the reasons of missing: missing completely at random (MCAR), where missing occurs completely at random and not related to any study variable; missing at random (MAR), where the probability of missing is related to the observed data; and missing not at random (MNAR), where the probability of missing depends on the unobserved data [2]. Common approaches for handling the missingness includes regression [3], maximum likelihood estimation [4,5], Bayesian methods [6], and multiple imputation (MI) [7,8]. Among these methods, multiple imputation is recognized for its ability to produce less bias and is a widely accepted approach that mitigates the impact of missing data in clinical research [9,10,11,12].

In this study, we assume missing at random (MAR), where missingness is conditionally independent of unobserved data given the observed covariates. This assumption underlies most multiple imputation methods, including those implemented here. However, it is important to acknowledge that missing not at random (MNAR) may occur in clinical datasets. For example, certain biomarkers may be more likely to be missing in patients with worse prognosis due to sample processing failures or selective testing, which may lead to a missingness pattern related to unobserved health status. While handling MNAR typically requires additional assumptions or sensitivity analyses, we focus here on MAR as a practically reasonable and widely used working assumption in oncology studies.

Penalized selection methods, such as the least absolute shrinkage and selection operator (LASSO) [13], adaptive least absolute shrinkage and selection operator (ALASSO) [14], and elastic net [15] have been extensively used to identify important predictors of clinical outcomes. However, when applied to multiply imputed datasets, these approaches pose a new challenge. In MI, each imputed dataset is a plausible version of the original data, and implementing variable selection separately to each one may yield inconsistent sets of selected variables. This inconsistency violates the assumptions under Rubin’s rules (RR) [7], making it difficult to combine estimates or draw overall conclusions.

Wood et al. [16] proposed three general strategies: (1) performing variable selection within each imputed dataset and selecting variables based on their inclusion frequency, (2) stacking all imputed datasets into a single dataset and applying variable selection once, using appropriate weights, and (3) conducting stepwise model selection using the Wald statistic combined across imputations via the RR. Although the RR approach was highly recommended for preserving the type I error rate, it was computationally intensive and may not scale well to larger datasets. However, Wood et al. mainly focused on continuous outcomes and assumed MCAR under most simulation scenarios, which limits the applicability of their findings in more realistic settings.

Subsequent research has extended penalized methods such as LASSO and elastic net to MI data, mostly for continuous or binary outcomes. Approaches include MI-LASSO, a group LASSO method [17] for joint modeling across imputed data, MI-WENet, a weighted elastic net method applied to stacked MI data [18], penalized objective functions that enforce consistent selection across imputations, with both “stacked” and “grouped” strategies [19], and variable selection based on the magnitude of estimates across imputations [20]. Building on earlier work, Zhao and Long [21] categorized imputation-based variable selection into pooled, stacked, and resampling-enhanced strategies, and highlighted that choice of method remains context-dependent and under-developed. Thao and Geskus [22] systematically compared LASSO-based approaches under multiple imputation and highlighted the robustness of the 1-SE penalty across settings.

Despite recent advances, variable selection with MI in survival analysis is underexplored. To our knowledge, studies applying group LASSO, inclusion frequency-based methods, or stacked penalization regression methods to the proportional hazards models under MAR are lacking. Recent work on more complex survival models—such as multi-parameter regression [23]—assume fully observed data, while earlier studies, such as Vonta et al. [24], used MI in survival modeling with AIC-based selection, but did not consider modern penalized regression techniques. Furthermore, the performance of penalized methods under criteria such as AIC or BIC remains understudied.

Our study addresses a critical gap by systematically evaluating penalized variable selection strategies in the context of survival analysis with MI data. Using data from a randomized phase III trial in men with metastatic castrate-resistant prostate cancer (mCRPC) (CALGB 90401) [25], we aimed to build a prognostic model of overall survival (OS) incorporating clinical and biomarker variables. The key challenge was the presence of missing baseline clinical covariates, which were assumed to be MAR. The MAR assumption was considered reasonable because missingness in these variables was likely associated with observed characteristics (e.g., age, performance status), rather than the unobserved values themselves. This aligns with the trial’s standardized study conduct, where missingness is often due to administrative reasons rather than underlying patient health status [9]. Building on methods proposed for continuous and binary outcomes, we adapt and assess the inclusion frequency and the stacked-data approaches—extending the framework of Wood et al. to penalized Cox models under realistic missing data mechanisms. Our contribution is primarily empirical and pragmatic: we evaluate and compare existing penalized approaches for variable selection under multiple imputation through extensive simulation and application to clinical trial data. We aim to determine the optimal strategy to perform penalized variable selection methods using MI data with survival outcomes, especially when the data structure and missing mechanism is similar to the CALGB 90401 data. To our knowledge, this is one of the first comprehensive evaluations of such approaches in the context of survival outcomes with MI.

The remainder of this article is organized in the following outline: the proposed variable selection methods are introduced in Section 2. The performance of the variable selection methods is then evaluated through extensive simulation studies and presented in Section 3. The variable selection methods are then applied to a real dataset in men with mCRPC in Section 4. Conclusions and discussions derived from both the simulation results and real-life application are presented in Section 5 and Section 6.

## 2. Methods

### 2.1. Penalized Models

We explored using LASSO and ALASSO penalty functions with different selection of tuning parameter λ: Akaike’s Information Criteria (AIC) [26], Bayesian Information Criteria (BIC) [27], minimum mean cross-validated error (CV.min), and within one standard error from the minimum (CV.1se). These penalized methods were chosen for their sparsity property [13], which shrinks weaker coefficients to zero to improve model interpretability, while also enhancing predictive accuracy. Moreover, penalized regression methods such as LASSO are well-established tools for reducing overfitting, as they effectively balance model fit with parameter shrinkage [28,29]. In our implementation, the tuning parameter λ was selected independently within each imputed dataset for all criteria (AIC, BIC, CV.min, and CV.1se) following standard practice in the MI context.

### 2.2. Model Selection Approaches with MI Data

We investigated three main model selection approaches using variable selection on each MI data separately and using variable selection on stacked MI data. We compared these proposed model selection strategies integrated with MI with the complete cases analysis (CC).

#### 2.2.1. Perform LASSO/ALASSO Selection on Each MI Data Separately

- AVG1: Select variables that are selected in any of the MI datasets
- AVG50: Select variables with an inclusion frequency >50% across MI datasets
- AVG70: Select variables with an inclusion frequency >70% across MI datasets
- AVG90: Select variables with an inclusion frequency >90% across MI datasets
- AVGALL: Select variables that are consistently selected by all MI datasets

For all the strategies above, the penalized model was fitted to each of the *M* datasets, where *M* denotes the number of imputations. The final variable selection was determined by the inclusion frequency of each variable across the *M* models. A ridge regression proportional hazards model was fitted to each of the *M* dataset using the selected variables and the regression coefficient estimates were averaged over all *M* models.

#### 2.2.2. Perform LASSO/ALASSO Selection on the Stacked Long Data

In the stacked approaches, all the *M* MI datasets were combined into a long single dataset, and a penalized model was fitted with weight assigned to each observation. *w_i_* denotes the weight for each observation in the stacked data.

- STK1: Assigned a uniform weight *w_i_* = 1/*M* to each observation, resulting in a total weight of 1 per individual across the M imputations
- STK2: Assigned weight *w_i_* = *f_i_*/*M* where *f_i_* was defined as the ratio of the number of complete variables for individual *i* to the total number of covariates, which is used previously in MI-WENet by Wan et al. [18]. This approach gives lower weight to individuals with more missing data.

For both stacked methods, a ridge penalized proportional hazards model was fitted to the stacked dataset using the previously selected variables to obtain the coefficient estimates.

#### 2.2.3. Perform Group LASSO/ALASSO Selection on Column-Bound Wide Data

To impose consistent selection across imputations, we implemented a group penalized approach by combining the *M* imputed datasets into a single wide-format dataset, where each covariate had *M* copies (one per imputation). A group LASSO (or group ALASSO) Cox model [30] was then fit, with the same covariate across imputations treated as a group and penalized jointly. This encourages selection consistency by either retaining or excluding the covariate in all imputations simultaneously.

To ensure comparability across methods, we adopted the following procedure: (i) Variable selection using the group penalized Cox model; (ii) Refitting the selected variables via ridge regression within each multiply imputed dataset, followed by averaging the refitted coefficients across imputations.

For each method described above, coefficient estimates from refitted ridge penalized regression models were averaged across imputations to provide a descriptive summary of variable selection and effect size. Because penalized models do not yield well-defined standard errors and the selected model may vary across imputations, Rubin’s rules could not be applied directly here. Accordingly, the average coefficients should be interpreted descriptively rather than inferentially. In simulation analysis, a sensitivity analysis was added to evaluate the empirical coverage of confidence intervals.

### 2.3. Evaluation of Different Approaches

The performance of the different approaches in the simulation studies was evaluated in terms of variable selection, parameter estimates, and predictive performance. The time-dependent area under the curve (tAUC) was also used to assess the predictive accuracy of the models [31]. For each simulation dataset, an independent testing set of 2000 individuals was generated under the same settings. The tAUC was then computed using the average parameter estimates from the refitted penalized proportional hazards model across *M* imputations [32].

In addition, we computed the integrated Brier score (IBS), calibration slope, and calibration intercepts at the 25th, 50th, and 75th percentiles of the observed event times, to evaluate overall prediction error and model calibration.

To evaluate the performance of the approaches when applied to the CALGB 90401 data in Section 4, as external validation data were not available, we calculated the optimism-corrected tAUC using bootstrap resampling. Specifically, for each of the M-imputed dataset, 200 bootstrapping samples were drawn. For each bootstrap sample, we computed the tAUC on the bootstrap sample (resampled data). The optimism was defined as the difference between the tAUC of the resampled and the original imputed data, and corrected tAUC was obtained by subtracting the optimism from the original tAUC on each MI data. These corrected tAUC values were summarized across imputations to assess model performance.

#### 2.3.1. Variable Selection

Our goal was to achieve a higher proportion of correct selections, positive discoveries, along with lower proportion of false positives and false negatives. Ideally, better performing models would identify more true covariates while excluding irrelevant (noise) covariates. We assessed each method’s ability to detect true covariates in the presence of noise, with performance metrics averaged across *S* simulation datasets to evaluate variable selection.

*Correct selection (CS)* was defined as the proportion of selecting exactly all the true covariates out of all simulation datasets.

(1) $$CS=\frac{1}{S}\sum_{S=1}^{S}\left\{\begin{array}{c} 1\;if\;exactly\;9\;true\;covariates\;are\;selected\\ 0\;otherwise\; \end{array}\right.$$

*Positive discovery (PD)* was defined as the average proportion of selecting the true covariates out of all selected covariates across all simulation datasets.

(2) $$PD=\frac{1}{S}\sum_{S=1}^{S}\frac{\#selected\;true\;covariates}{\#\;all\;selected\;covariates}$$

*False positive (FP)* was calculated as the average proportion of noise covariates selected by the model out of all noise covariates.

(3) $$FP=\frac{1}{S}\sum_{s=1}^{S}\frac{\#selected\;noise\;covariates}{\#\;all\;noise\;covariates}$$

*False negative (FN)* was calculated as the average proportion of the true covariates not selected by the model out all true covariates.

(4) $$FN=\frac{1}{S}\sum_{s=1}^{S}\frac{\#true\;covariates\;not\;selected}{\#\;all\;true\;covariates}$$

#### 2.3.2. Parameter Estimates

The parameter estimates of the models were assessed using the following criteria and the final values were averaged across the nine true covariates and *S* simulation data, with $\hat{\beta }$ defined as the estimated coefficient and β as the true coefficient:

*Average bias*:

(5) $$BIAS=\frac{1}{S}\sum_{s=1}^{S}(\beta -\hat{\beta })$$

*Mean Squared Error (MSE*):

(6) $$MSE=\frac{1}{S}\sum_{s=1}^{S}(\beta -\hat{\beta }{)}^{2}$$

## 3. Simulation

### 3.1. Simulation Design

Simulation studies were conducted to compare the performance of each variable selection method, motivated by the CALGB 90401 randomized phase III trial. Each simulated dataset included 500 individuals with 20 covariates denoted as ***X***(*X*_1_ − *X*_20_) with *X*_1_ − *X*_9_ designated as true covariates and *X*_10_ – *X*_20_ as noise. The number of covariates was chosen to reflect the number of covariates considered for building the prognostic model of OS in prostate cancer. Covariates were generated from a multivariate normal distribution with mean 0, unit variance, and pairwise correlation of 0.1 to mimic realistic correlation structures. In addition, *X*_2_, *X*_3_ and *X*_5_ were dichotomized at 0 to create binary variables (values greater than or equal to zero coded as 1, and values less than 0 as 0).

The survival time was simulated from the proportional hazards model using all the true covariates (*X*_1_ − *X*_9_) with the following hazard function:

(7) $$h(t|X)=h_{0}(t)\exp(X^{T}\beta ),$$

where $\beta =(\beta_{1},\beta_{2},\beta_{3},\beta_{4},\beta_{5},\beta_{6},\beta_{7},\beta_{8},\beta_{9})$.

The baseline hazard function *h*_0_(*t*) was assumed to follow a Weibull distribution (*κ* = 2, λ=0.001) [33,34], motivated by its widespread use in simulating Cox proportional hazards models and its consistency with the survival patterns observed in our motivating CALGB 90401 dataset. Censoring times were independently generated from an independent uniform distribution $\sim U\left(0,\theta \right),$ where the censoring parameter θ was selected to achieve the target 0.1 or 0.3 censoring proportions. These levels reflect what was observed in the CALGB 90401 study (censoring proportion = 0.1) and are consistent with other oncology studies [35,36,37]. Following our prior work [38], we considered two sets of regression coefficients to represent weak and strong signals; the following scenarios were considered.

The coefficients for the weak signal were $\beta_{1}=0.375,\beta_{2}=-0.5,\beta_{3}=0.75,\beta_{4}=-0.375,\beta_{5}=0.75,\beta_{6}=0.5,\beta_{7}=0.25,\beta_{8}=-0.75,\beta_{9}=0.5$, whereas the coefficients for the strong signal were $\beta_{1}=2.38,\beta_{2}=-2.02,\beta_{3}=-2.19,\beta_{4}=-2.26,\beta_{5}=-2.00,\beta_{6}=-2.25,\;\beta_{7}=2.11,\beta_{8}=-2.33,\beta_{9}=2.27.$

MAR was implemented, consistent with the strong plausibility that missingness in our study arose from observed clinical factors rather than from unmeasured baseline variables [10,39]. In randomized clinical trials, missing baseline covariates are often related to characteristics such as age, study center, or performance status, and not the unobserved values themselves. To simulate this mechanism, a logistic regression model was used for the probability of missingness for covariate *X_j_*, where *j* = 2,4,12,14 based on complete covariates ***X_c_*** = (*X*_1_, *X*_3_, *X*_5_, *X*_7_, *X*_8_, *X*_9_). The probability of missingness *R_ij_* for individual *i* and variable *j* for j = 2,4,12,14 was defined as:

(8) $$Logit(Pr(R_{ij}|X_{c})=\alpha_{0}+0.25X_{ic}^{T}.$$

The value of *α*_0_ is then selected to achieve 10% and 20% of missingness, respectively. Importantly, the missing mechanism was implemented independently of the censoring mechanism in all simulations. That is, the probability of a covariate being missing was not influenced by whether or when a participant was censored. We explored various combinations of censoring proportion (*C*) (at 0.1 and 0.3), proportions of missing values per covariate (*pm*) (at 10% and 20%), and the number of multiple imputations (*M*) (at 10 and 30). The maximum missingness observed in the baseline covariates in the CALGB 90401 dataset was 19.1% for opioid analgesic use, with BMI at 12.4% and most other baseline variables ≤0.5%. Thus, simulating 10–20% missingness allowed us to reflect the level of incompleteness in our dataset while also covering an upper yet realistic level commonly encountered in clinical trials [40,41]. Due to the extensive computing time, 100 simulation datasets (*S*) of 500 individuals were created under each of the eight scenarios, with the run time of approximately 8–12 h for each task. MI was performed using the {mice} package in R [42], which assumes that missingness is at random (MAR). The assumption is consistent with the design of the CALGB 90401 trial. The MI process utilized random-forest method (meth = “rf”, ntree = 10), generating *M* = 10 or *M* = 30 completed datasets according to the scenario. Before imputation, we approximated the cumulative hazard to the survival time using the Nelson–Aalen estimator and included this variable as a predictor in the imputation model, following the recommendation of White and Royston to carry out survival information into the imputation step [43].

### 3.2. Sensitivity Analysis

Because penalized selection followed by refitting within each imputed dataset does not directly provide standard errors, we conducted a focused sensitivity analysis to examine CI coverage for the three configurations that are most representative of our proposed workflows: AVG50, STK2, and GRP with ALASSO and BIC tuning. For the CI sensitivity analysis, after the adaptive group LASSO selected a set of predictors, we recomputed robust standard errors using an event time-based sandwich estimator for the Cox partial likelihood. The estimator aggregates score and risk-set information across all failure times, adjusts the information matrix on the active (nonzero) coefficients to account for the adaptive penalty, inverts the adjusted matrix, and maps it back to the full parameter vector. Wald-type 95% CIs were then formed from the diagonal of this sandwich covariance. We evaluated empirical CI coverage in the weak signal, high-missingness, high-censoring scenario (MI = 10, pm = 30%, C = 0.30) using:

*Mean CI Coverage (CI.Cov):*

(9) $$\begin{array}{c} Mean\;Coverage\\ =\frac{1}{S}\sum_{s=1}^{S}\begin{array}{c} 1\;if\;the\;true\;coefficient\;is\;covered\;by\;the\;estimated\;95\%\;CI\\ 0\;otherwise\; \end{array}, \end{array}$$

and the reported coverage was averaged over the *S* simulation replicates and over the MI multiply imputed datasets. Here, for the sensitivity analysis *S* = 100 replicated simulation dataset.

### 3.3. Simulation Results

#### 3.3.1. Weak Signal

**Figure 1:**
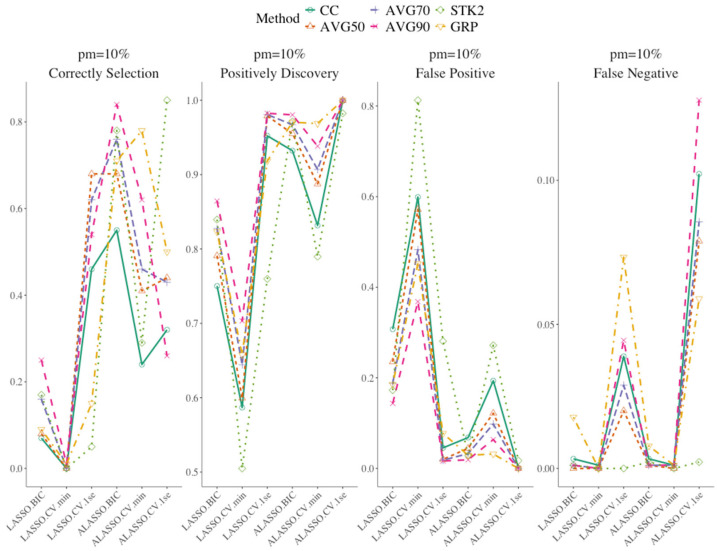
Summary variable selection results using weak signal, 10 multiple imputation and 0.10 censoring, 10% missing.

Figure 1 presents the results of the variable selection under the weak signal setting, with 10 imputed data (*M*), 10% missing (*pm*), and 0.10 censoring (*C*). Under this certain combination of *M*, *pm*, and *C*, ALASSO BIC with moderate inclusion frequency (AVG50, AVG70) gave the most balanced variable selection performance, with consistently balanced sensitivity (capturing the true covariates) and specificity (limiting noise), whereas the 1SE tuned versions were noticeably more conservative and tended to miss borderline true predictors (Panel: Falso Negative). Similarly, the GRP approach tends to give the most stable predictors across imputations. It does not pick up a lot of noise, but at the price of missing true covariates. The stacked methods showed stability in retaining true covariates, but at the cost of slightly higher false positive rates compared with inclusion frequency approaches. It is worth noting that all approaches maintained low proportion of false negative.

Table 1 shows the summary statistics of the results from the simulation of the variable selection with 10 imputed data (*M*) and 0.1 censoring (*C*). When the percentage increased from 10% to 20%, ALASSO.CV.1se with 50% inclusion frequency remained top-performing method across all metrics. Additional variable selection results with various combination of number of imputed data, censoring and percentage missing are presented in the Supplementary Figures S1–S4. Among all factors, censoring had the highest impact on variable selection, when moving from *C* = 0.10 to *C* = 0.30. In contrast, increasing the number of imputations from 10 to 30 had minimal effect on variable selection when the percentage missing was moderate. When *C* = 0.3, the false negative proportion increased for most methods, reflecting the loss of information from fewer observed events, and thus reduced the effective information available for distinguishing between true predictors and noise. This pattern was most evident for stricter inclusion rules such as AVG90 and AVGALL, which require near consensus across imputations to retain a predictor. Stacked methods (STK1/STK2) continued to show selection stability—they tended to keep more true variables—but this came with modestly higher false positive rates than inclusion frequency-based methods.

Compared to other approaches, AVG1 selected more noise covariates and had a high proportion of false positive, as it retained any variable selected in at least one imputed dataset, leading to the inclusion of many noise covariates. In contrast, AVGALL, with its strict selection criterion, selected the fewest noise covariates. Both stacked approaches (STK1 and STK2) produced similar variable selection results. ALASSO with the BIC in stacked methods achieved higher proportions of correct selections and positive discoveries, while maintaining relatively lower false positive and false negative proportion. Overall, ALASSO with BIC using a moderate inclusion frequency gave the best trade-off between true variable selection and controlling false positives, while maintaining a low proportion of false positive, false negative, and high proportion of correct selection and positive discovery among all the variable selection strategies.

**Figure 2:**
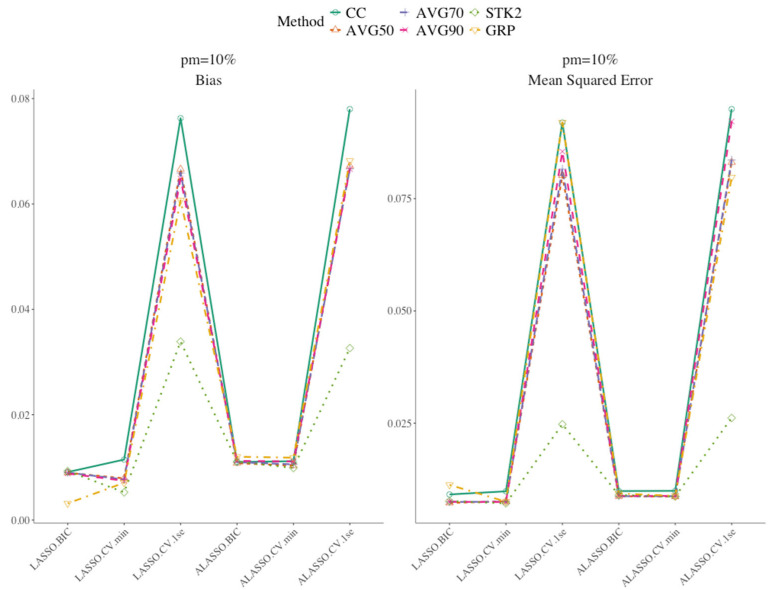
Summary parameter estimates results using weak signal, 10 multiple imputation and 0.10 censoring, 10% missing.

Figure 2 shows the summary statistics for the results of the parameter estimates simulation. Among all approaches CC had the highest bias and MSE. Table 2 presents the summary statistics of the parameter estimates for simulations with 10 imputed data and 0.1 censoring. Additional results for other combinations of imputation number, censoring rates, and missing percentage are presented in Supplementary Figures S5 and S6. No single method consistently outperformed the others across all scenarios. For most approaches, both bias and MSE increased as the proportion of missing data increased. When the proportion of the missing remained the same, the impact of *C* = 0.3 was similarly adverse as in variable selection: bias and MSE increased relative to *C* = 0.10 across most methods. Nevertheless, BIC and min lambda-based approaches continued to yield lower bias and MSE, with AIC close behind, preserving their advantage even as censoring rose, though the gap narrowed under heavier censoring and weaker signals. Increasing the number of imputations from 10 to 30 had a negligible impact on the parameter estimates. Overall, ALASSO and LASSO approaches with 1SE maintained relatively higher bias and MSE than the other penalized methods. These trends persisted with STK1 and STK2, although the differences between penalized methods were less pronounced under the stacked approaches. GRP approach was generally well behaved but not dominant. It kept bias in a reasonable range, yet its MSE was often slightly higher than its BIC-tuned AVG counterparts.

**Figure 3:**
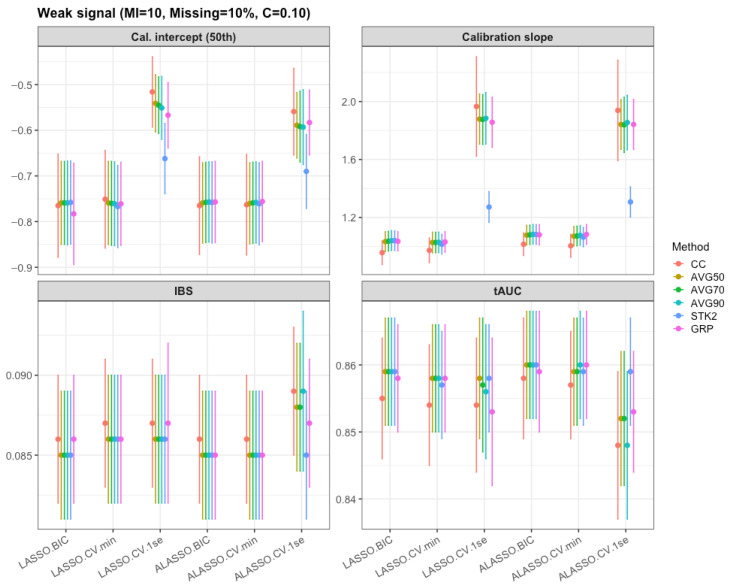
Mean tAUC, Integrated Brier score and calibration (95% CI) under the weak signal setting (10 multiple imputation, 0.10 censoring, and 10% missing).

Across all scenarios, differences in tAUC were small between methods, based on Figure 3, Table 3, and Supplementary Figure S7. The main drivers were data scarcity factors: moving from 0.1 to 0.3 censoring and from 10% to 20% missingness produced small, consistent declines in tAUC, while increasing the number of imputations from 10 to 30 had negligible impact. Notably, parsimonious choice (e.g., ALASSO with 1SE and moderate inclusion thresholds) achieved similar tAUC to more complex models such as min lambda, indicating that aggressive variable retention did not translate into significantly better tAUC here. In summary, under weak signals, modest losses in information (higher censoring or missingness) reduces tAUC slightly. Beyond discrimination, we also summarized the integrated Brier score (IBS), calibration slope, and calibration intercepts at the 25th, 50th, and 75th percentiles of follow-up for all methods and tuning rules. These results are displayed in Figure 3 and Figures S8–S12, which shows that IBS values were tightly clustered, especially across the inclusion threshold (AVG50–AVG90) strategies, indicating that none of the approaches introduced substantial additional prediction error. Calibration slopes were generally close to 1.0 for the averaging-based methods. The LASSO and ALASSO models tuned with the 1se rule showed noticeably larger calibration slopes since their stronger penalty over shrunk the coefficients, producing risk scores with low variability then required reinflation at the calibration stage. Taken together, the MI strategies that reduced cross-imputation selection variability (e.g., AVG50) also preserved overall accuracy and calibration, not just tAUC.

#### 3.3.2. Strong Signal

**Figure 4:**
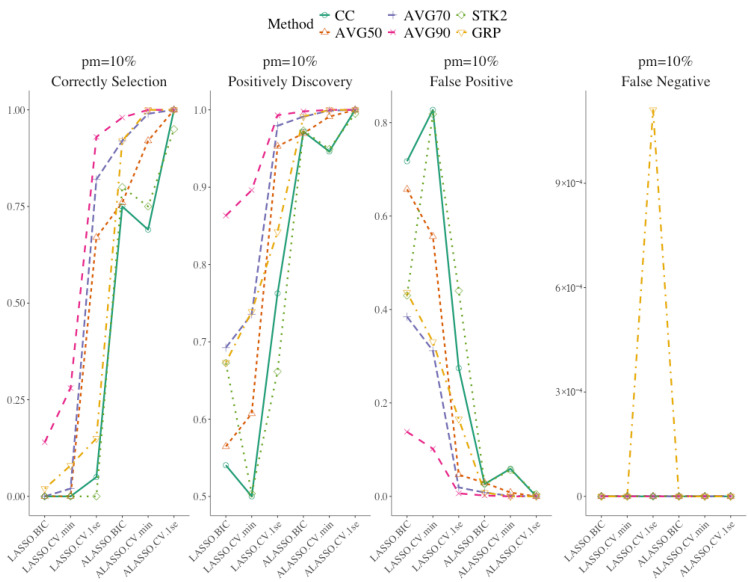
Summary variable selection results using strong signal, 10 multiple imputation and 0.10 censoring, 10% missing.

Figure 4 displays the summary of the results of the variable selection for the strong signal setting (10 imputed data, 10% missing, and 0.1 censoring). ALASSO with 1SE and moderate inclusion frequency now achieving the best balance of correct selection, positive discovery, and low false positive proportion. Compared to the weak signal setting, all the methods tended to have a lower proportion of false negative and false positive, since the true covariates are easier to detect. ALASSO with minimal lambda and ALASSO with BIC had improved variable selection under the strong signal, with increases in correct selection, positive discovery, and false positive rates. Table 4 presents detailed statistics, and Supplementary Figures S11–S15 provide additional combinations of settings. Under strong signal, both false negative and false positive decreased because larger effects are easier to detect and spurious inclusions are less likely. In *C* = 0.30 setting, variable selection decreased less than in weak-signal settings, and ALASSO with 1SE and moderate inclusion thresholds again balanced sensitivity and specificity well. ALASSO models with BIC and min lambda exhibited improved correct selection and positive discovery rates under strong signals. Stacked approaches remained competitive but retained relatively higher false positives than the inclusion frequency-based approaches. Similarly to the results observed earlier in weaker setting, the false positive rate decreased from AVG1 to AVGALL, and ALASSO models with BIC, min, and 1SE lambdas continued to show strong performance. Although ALASSO 1SE with AVGALL and GRP with LASSO 1SE showed slightly higher false negatives under certain scenarios, the magnitude was minimal.

**Figure 5:**
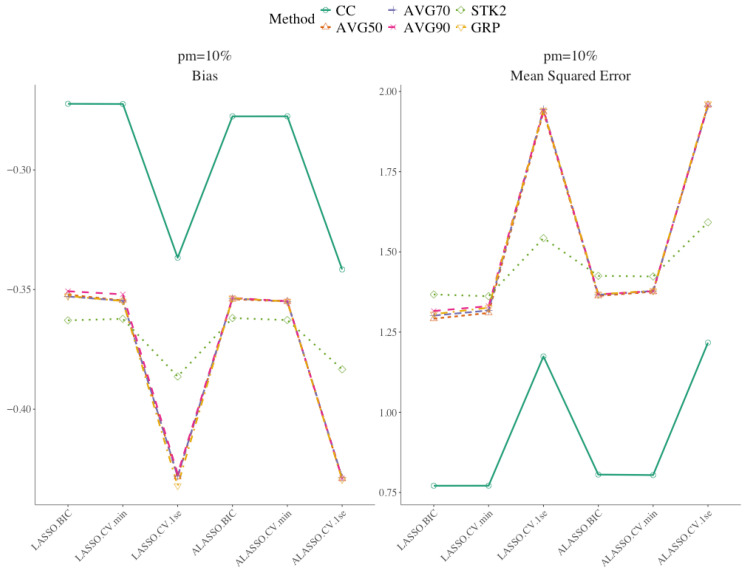
Summary parameter estimates results using strong signal, 10 multiple imputation and 0.10 censoring, 10% missing.

Figure 5 presents the parameter estimates results (10 imputed data, 10% missing, and 0.1 censoring). Overall, bias and MSE were higher than the weak signal settings, an expected finding due to the larger true values of the coefficients. Table 5 shows the summary statistics of parameter estimate results and Supplementary Figures S17 and S18 provide additional combination settings. AIC, BIC, and min lambda approaches continued to deliver the lowest bias and MSE across censoring levels, including *C* = 0.30. 1SE approaches tended to be more conservative and showed slightly higher bias and MSE. Stacked approaches produced moderate estimation errors with little separation between stacking weights. For both signal strengths, stacked methods tend to emphasize stability—capturing more true variables, but at the cost of slightly higher false positives.

**Figure 6:**
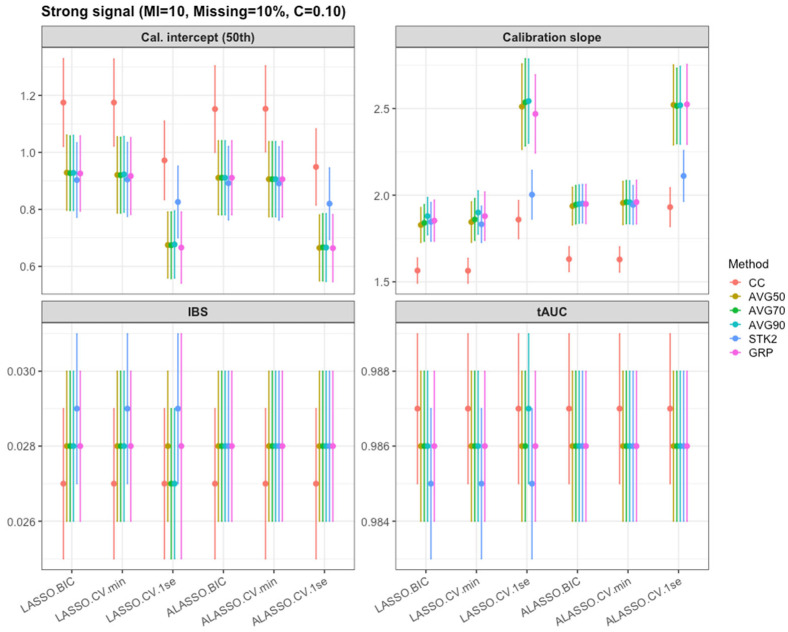
Mean tAUC, Integrated Brier score and calibration (95% CI) under the strong signal setting (10 multiple imputation, 0.10 censoring, and 10% missing).

With stronger effects, overall tAUC levels were slightly higher and remained remarkably stable across penalization and selection strategies, as shown in Table 6 and Supplementary Figure S19. Even under heavier censoring (0.3) or higher missingness (20%), the reductions in tAUC were small. More complex models such as min lambda did not yield detectable gains in tAUC over more conservative 1SE or BIC selections. Stacked versus inclusion-frequency strategies showed comparable discriminative performance. In summary, in strong signal settings, tAUC is largely insensitive to the modeling variant. A similar pattern was seen for the integrated Brier Score in Figure 6. Point estimates were tightly clustered across methods and tuning choices and increases in missingness or censoring led to only modest degradations in overall prediction error. Calibration measures were somewhat more method-sensitive: LASSO/ALASSO models tuned by the 1SE rule tended to have larger calibration slopes and shifted intercepts, consistent with their slightly over-shrunk risk scores. Under strong signals, the main methods deliver comparable Brier Scores, and differences are driven primarily by how aggressively the penalty shrinks the linear predictor.

In the sensitivity analysis, the mean CI coverage was reported in Supplementary Table S1. The CIs showed the expected under-coverage, especially for weaker signal with high missingness. Mean CI coverage was the highest for AVG50 approach, lower for GRP, and poorest for STK2, which is consistent with STK2′s more aggressive down-weighting across imputations.

## 4. Application

We analyzed data from CALGB 90401, a phase III trial of 1050 men with mCRPC comparing docetaxel plus prednisone with either bevacizumab (DP+B) or placebo (DP) [25]. The primary outcome of the study was OS, defined as the time from date of random assignment to date of death or last follow-up. We had previously developed and validated a prognostic model for predicting overall survival for mCRPC patients using this dataset addressing missing data via regression imputation [44].

For the current analysis, we have focused on the 853 patients who consented to plasma and serum collection, incorporating eight clinical predictors of OS from our previous model: site of metastasis disease (bone metastases only (DS2), any visceral metastases (DS3)), opioid analgesic use (PAIN), Eastern Cooperative Oncology Group performance status (ECOG), LDH > 1 upper limit of normal (LDH.High), albumin (ALB), hemoglobin (HGB), alkaline phosphatase (ALKPHOS), and prostate-specific antigen (PSA) [44]. We considered to expand the model to include 24 plasma angiokines (Ang-2, BMP-9, CD-73, Chromogranin A, HER-3, HGF, ICAM-1, IL-6, OPN, PDGF-AA, PDGF-BB, PIGF, SDF-1, TGF-b1, TGF-b2, TGFb-R3, TIMP-1, TSP-2, VCAM-1, VEGF, VEGF-D, VEGF-R1, VEGF-R2, and VEGF-R3) and 3 serum androgens (testosterone, androstenedione, and dehydroepiandrosterone) aiming to increase the prognostic accuracy of the OS model. Proportional hazards assumptions for the core clinical predictors (DS2, DS3, ECOG, LDH.High, ALB, HGB, ALKPHOS) were evaluated using Schoenfeld residuals. 

Among the 853 consenting patients, opioid analgesic use had the highest missingness (19.1% missing) and 538 patients (62.6%) had complete information for all the eight clinical variables (Table 7). To provide baseline survival characteristics, we include the Kaplan–Meier curve for overall survival in the CALGB 90401 data (Supplementary Figure S25) and a complete-case Cox regression summary (Supplementary Table S2). We created 10 MI datasets and assessed the model performance by examining the covariates selected and the average coefficient from refitted penalized models. The summary variable selection results and corresponding coefficient estimates across the various methods are presented in Table 8. In general, CC selected the fewest variables, while STK2 tended to select the most variables. GRP with ALASSO cv and 1SE did not select any variables. Among the penalized approaches, LASSO/ALASSO with minimal lambda selected more variables than 1SE and BIC, although some of the selected variables had small coefficient estimates with hazard ratios (HR) close to one. The three serum androgens were either not selected or had HRs close to one when included, suggesting limited prognostic value. Among the 24 plasma angiokines, ICAM-1, TIMP-1, and VEGF-R3 were selected by a few approaches (such as with LASSO/ALASSO with minimal lambda and 70% inclusion frequency) and showed relatively larger HR. This suggested that these angiokines may have potential prognostic value for overall survival in the mCRPC population studied in CALGB 90401. All the methods produced comparable tAUC values after correcting for optimism. Model performance showed slight improvement as more covariates were selected (from AVG90 to AVG50), although these differences were modest in magnitude (Table 9). Across the MI-averaging and stacking strategies, IBS values (Table 10) were very similar. Calibration slopes (Table 11) for most approaches were close to one, indicating no gross miscalibration. These findings are consistent with the simulation results.

## 5. Discussion

In this article, we evaluated and compared the performance of various variable selection methods in combinations with MI, a widely accepted and used approach for handling missing data in clinical studies. MI creates several plausible complete datasets, applies standard statistical methods to each imputed dataset, and then combines the resulting estimates. This approach incorporates the variability arising from both the imputation and the estimation process, thereby reflecting the true uncertainty associated with missing data. MI is very efficient and achieves maximal data usage and reduces the potential risk of bias inherent in complete cases studies. However, the combination of MI and variable selection introduces a substantial layer of complexity into the development of prognostic models. Variable selection is inherently unstable in the presence of missing data, and MI, by design, produces multiple versions of the dataset. Combining the outputs of multiple selection processes—each potentially yielding a different set of variables—requires careful methodological consideration. This complexity is not unique to our study but is an unavoidable issue when building prognostic models in real-world datasets, particularly in oncology, where missingness is often non-trivial.

Our simulations demonstrated that no single method consistently performed the best under all circumstances, whether in terms of variable selection, or the parameter estimates under both weak and strong signal settings. In strong signal settings, all methods tended to have lower proportions of false negative and false positive, a pattern consistent with the expectation that the existence of a stronger signal makes true covariates easier to identify while reducing spurious selections. This improvement reflects greater stability in selection. Some methods—particularly the penalized regression approaches (LASSO/ALASSO) with minimal lambda—often selected more variables than their 1SE or BIC-tuned counterparts. This improvement in sensitivity often came at the cost of including additional variables with weaker effects, some of which had hazard ratios close to one, indicating limited clinical relevance. The inclusion of predictors with small effect sizes raises the question of whether such expanded models meaningfully improve predictive performance. In fact, the optimism-corrected tAUC values across methods were remarkably similar, suggesting that model parsimony may not substantially compromise predictive accuracy in this context. The consistently higher bias and MSE observed for the 1SE approaches (for both LASSO and ALASSO) are expected. The 1SE rule selects a larger penalty than min lambda, yielding sparser models and greater shrinkage of non-zero coefficients toward zero—i.e., increased shrinkage bias. Under MI, this stronger penalty also raises the chance that weak but truly associated covariates are excluded in some imputations. After aggregation (e.g., by inclusion frequency threshold), some of these true covariates are dropped, introducing omitted-variable bias. This conservatism also reflects as slightly higher false negatives than AIC, BIC, or min lambda in variable selection, particularly when *C* = 0.30. Therefore, even though 1SE yields well-balanced variable selection results with fewer false positives, the combination of heavier shrinkage and occasional exclusion of weak signals increased bias and MSE relative to AIC, BIC, and min lambda.

There are a few limitations that warrant consideration. First, our simulations assumed an MAR mechanism. While we think this is a reasonable assumption for CALGB 90401, alternative mechanisms, such as MNAR, could affect the performance of these approaches. Second, our simulations were limited to specific conditions for the proportion of missingness, level of censoring, and the number of imputations. These design choices were guided by the characteristics of the CALGB 90401 trial, which may limit the generalizability of our findings to substantially different settings. We used a Weibull distribution to simulate event times because it is commonly employed in survival simulation studies and it closely approximated the empirical survival distribution observed in CALGB 90401 and other cancer studies [45]. Third, a key limitation of our approach is the lack of formal variance estimation when combining post-selection penalized models across imputations. Since penalized regression does not yield valid within-imputation standard errors, and the selected variables may differ across imputations, Rubin’s rules could not be applied. As such, the averaged coefficients presented should be interpreted as descriptive rather than inferential. While we attempted to address this limitation through empirical coverage evaluation in simulation, further methodological development can be conducted to provide valid inference after penalized model selection under multiple imputation. An additional limitation of our study lies in the independent selection of tuning parameters across imputations. While we used a conventional approach where λ is tuned separately within each imputed dataset, this can exacerbate variability in selected models across imputations and contribute to reduced selection stability. Coordinated tuning strategies, such as selecting a single λ across imputations based on pooled information or stacking techniques, may improve consistency and warrant further investigation. Another limitation concerns the STK2 approach, which uses a simple completeness-based weighting scheme. While this method, adopted from MI-WINet by Wan et al., is computationally convenient, it does not account for the relationship between missingness and observed covariates, which could bias results under more complex missing data mechanisms. Alternative model-based weighting strategies, such as inverse probability weighting based on a fitted missingness model, could be explored in the future to more appropriately address the missing data mechanism under MAR or MNAR. Due to computational constraints, optimism correction was applied only to the tAUC via bootstrap resampling. The Integrated Brier Score (IBS) and calibration metrics (slope and intercepts) were computed on the original imputed data without optimism correction. While this limits direct comparability across metrics, these additional measures were included to provide complementary insight into overall prediction error and calibration. We acknowledge this as a limitation and note that future work could extend optimism correction to all metrics for consistency. Beyond variable selection and estimation accuracy, we evaluated predictive performance using time-dependent AUC, integrated Brier score, and calibration indices to capture discrimination, overall prediction error, and calibration. While additional decision-oriented metrics such as risk stratification performance or decision curve analysis could offer further insight into clinical utility, their added modeling and computational demands under the multiply imputed setting were therefore beyond our current scope. We therefore view these extensions as valuable future work aimed at assessing the clinical utility of MI-based selection and prediction strategies.

Finally, this work is intended to provide pragmatic guidance for applied researchers, rather than new theoretical guarantees. Our findings are based on empirical evaluation of methods under realistic conditions, and do not establish formal consistency or optimality properties. While our simulation provides useful empirical insights, there remains limited formal theory supporting variable selection consistency or risk bounds in the setting of penalized Cox models with multiple imputations. Development of such theoretical foundations represents an important direction of future work.

## 6. Conclusions

Our findings underscore that there is no universally “best” method for variable selection in the context of MI and survival data. Method performance depends on signal strength, the degree of missingness, level of censoring, and the underlying correlation structure of the predictors. Nonetheless, certain approaches demonstrated consistently competitive performance in specific scenarios and may serve as strong candidates for use in similar prognostic modeling settings. For clinical researchers, the key message is that the choice of method should be driven not only by statistical performance metrics but also by considerations of model interpretability, parsimony, and clinical utility.

## Figures and Tables

**Table 1:** Summary Statistic of variable selection by simulation settings for weak signal.

	Missing = 10%, MI = 10, Censoring = 0.10						Missing = 20%, MI = 10, Censoring = 0.10					
	CC	AVG50	AVG70	AVG90	STK2	GRP	CC	AVG50	AVG70	AVG90	STK2	GRP
Correctly Selection												
LASSO.BIC	0.07	0.08	0.16	0.25	0.17	0.09	0.07	0.14	0.18	0.28	0.18	0.07
ALASSO.BIC	0.55	0.68	0.76	0.84	0.78	0.71	0.41	0.67	0.73	0.82	0.77	0.73
LASSO.CV.min	0.00	0.00	0.00	0.01	0.00	0.01	0.00	0.00	0.01	0.02	0.00	0.00
ALASSO.CV.min	0.24	0.41	0.46	0.62	0.29	0.78	0.19	0.37	0.45	0.56	0.24	0.60
LASSO.CV.1se	0.46	0.68	0.62	0.54	0.05	0.15	0.13	0.54	0.51	0.37	0.06	0.14
ALASSO.CV.1se	0.32	0.44	0.43	0.26	0.85	0.50	0.15	0.28	0.21	0.11	0.81	0.52
Positively Discovery												
LASSO.BIC	0.75	0.79	0.83	0.87	0.84	0.82	0.76	0.81	0.85	0.89	0.86	0.83
ALASSO.BIC	0.93	0.96	0.97	0.98	0.97	0.97	0.93	0.96	0.97	0.98	0.97	0.98
LASSO.CV.min	0.59	0.60	0.64	0.70	0.51	0.66	0.62	0.61	0.66	0.73	0.50	0.64
ALASSO.CV.min	0.83	0.89	0.91	0.94	0.79	0.97	0.84	0.88	0.91	0.94	0.78	0.94
LASSO.CV.1se	0.95	0.98	0.98	0.98	0.76	0.92	0.95	0.98	0.98	0.98	0.77	0.93
ALASSO.CV.1se	1.00	1.00	1.00	1.00	0.98	1.00	1.00	1.00	1.00	1.00	0.98	1.00
False Positive												
LASSO.BIC	0.31	0.24	0.19	0.14	0.17	0.19	0.29	0.21	0.16	0.11	0.15	0.17
ALASSO.BIC	0.07	0.05	0.03	0.02	0.03	0.03	0.07	0.04	0.03	0.02	0.03	0.02
LASSO.CV.min	0.60	0.57	0.48	0.37	0.81	0.45	0.52	0.56	0.46	0.33	0.82	0.49
ALASSO.CV.min	0.19	0.12	0.10	0.06	0.27	0.03	0.18	0.13	0.09	0.06	0.29	0.06
LASSO.CV.1se	0.05	0.02	0.02	0.02	0.28	0.08	0.05	0.02	0.02	0.02	0.26	0.07
ALASSO.CV.1se	0.00	0.00	0.00	0.00	0.02	0.00	0.00	0.00	0.00	0.00	0.02	0.00
False Negative												
LASSO.BIC	0.00	0.00	0.00	0.00	0.00	0.02	0.02	0.00	0.00	0.00	0.00	0.02
ALASSO.BIC	0.00	0.00	0.00	0.00	0.00	0.01	0.03	0.00	0.00	0.01	0.00	0.01
LASSO.CV.min	0.00	0.00	0.00	0.00	0.00	0.00	0.01	0.00	0.00	0.00	0.00	0.00
ALASSO.CV.min	0.00	0.00	0.00	0.00	0.00	0.00	0.02	0.00	0.00	0.00	0.00	0.00
LASSO.CV.1se	0.04	0.02	0.03	0.04	0.00	0.07	0.12	0.04	0.05	0.07	0.00	0.08
ALASSO.CV.1se	0.10	0.08	0.09	0.13	0.00	0.06	0.20	0.12	0.15	0.20	0.01	0.06

**Table 2:** Summary Statistic of parameter estimates by simulation settings for weak signal.

	Missing = 10%, MI = 10, Censoring = 0.10						Missing = 20%, MI = 10, Censoring = 0.10					
	CC	AVG50	AVG70	AVG90	STK2	GRP	CC	AVG50	AVG70	AVG90	STK2	GRP
Bias												
LASSO.BIC	0.01	0.01	0.01	0.01	0.01	0.01	0.01	0.01	0.01	0.01	0.01	0.01
ALASSO.BIC	0.01	0.01	0.01	0.01	0.01	0.01	0.01	0.01	0.01	0.01	0.01	0.01
LASSO.CV.min	0.01	0.01	0.01	0.01	0.00	0.00	0.02	0.01	0.01	0.01	0.00	0.00
ALASSO.CV.min	0.01	0.01	0.01	0.01	0.00	0.00	0.01	0.01	0.01	0.01	0.00	0.00
LASSO.CV.1se	0.08	0.07	0.07	0.07	0.03	0.03	0.09	0.07	0.07	0.06	0.03	0.03
ALASSO.CV.1se	0.08	0.07	0.07	0.07	0.03	0.03	0.10	0.07	0.07	0.07	0.03	0.03
MSE												
LASSO.BIC	0.01	0.01	0.01	0.01	0.01	0.01	0.02	0.01	0.01	0.01	0.01	0.01
ALASSO.BIC	0.01	0.01	0.01	0.01	0.01	0.01	0.02	0.01	0.01	0.01	0.01	0.01
LASSO.CV.min	0.01	0.01	0.01	0.01	0.01	0.01	0.02	0.01	0.01	0.01	0.01	0.01
ALASSO.CV.min	0.01	0.01	0.01	0.01	0.01	0.01	0.02	0.01	0.01	0.01	0.01	0.01
LASSO.CV.1se	0.09	0.08	0.08	0.09	0.03	0.03	0.12	0.09	0.09	0.10	0.03	0.03
ALASSO.CV.1se	0.10	0.08	0.08	0.09	0.03	0.03	0.13	0.10	0.10	0.11	0.03	0.03

**Table 3:** Summary of tAUC (standard deviation) from ridge regression for the simulation with 10 multiple imputation using weak signal.

	LASSO.BIC	ALASSO.BIC	LASSO.CV.min	ALASSO.CV.min	LASSO.CV.1se	ALASSO.CV.1se
Missing = 10%, MI = 10, Censoring = 0.10						
CC	0.855 (0.009)	0.858 (0.009)	0.854 (0.009)	0.857 (0.008)	0.854 (0.01)	0.848 (0.011)
AVG50	0.859 (0.008)	0.86 (0.008)	0.858 (0.008)	0.859 (0.008)	0.858 (0.009)	0.852 (0.01)
AVG70	0.859 (0.008)	0.86 (0.008)	0.858 (0.008)	0.859 (0.008)	0.857 (0.01)	0.852 (0.01)
AVG90	0.859 (0.008)	0.86 (0.008)	0.858 (0.008)	0.86 (0.008)	0.856 (0.01)	0.848 (0.011)
STK2	0.859 (0.008)	0.86 (0.008)	0.857 (0.008)	0.859 (0.008)	0.858 (0.008)	0.859 (0.008)
GRP	0.858 (0.008)	0.859 (0.009)	0.858 (0.008)	0.86 (0.008)	0.853 (0.011)	0.853 (0.009)
Missing = 10%, MI = 10, Censoring = 0.30						
CC	0.847 (0.011)	0.849 (0.011)	0.846 (0.011)	0.848 (0.011)	0.834 (0.017)	0.817 (0.026)
AVG50	0.851 (0.01)	0.852 (0.01)	0.85 (0.01)	0.851 (0.01)	0.842 (0.016)	0.829 (0.018)
AVG70	0.851 (0.01)	0.852 (0.01)	0.85 (0.01)	0.852 (0.01)	0.841 (0.016)	0.827 (0.018)
AVG90	0.851 (0.01)	0.852 (0.01)	0.85 (0.01)	0.852 (0.01)	0.839 (0.017)	0.823 (0.018)
STK2	0.851 (0.01)	0.852 (0.01)	0.849 (0.01)	0.851 (0.01)	0.85 (0.01)	0.85 (0.011)
GRP	0.846 (0.013)	0.85 (0.012)	0.85 (0.01)	0.852 (0.01)	0.842 (0.013)	0.841 (0.013)
Missing = 20%, MI = 10, Censoring = 0.10						
CC	0.852 (0.01)	0.854 (0.009)	0.85 (0.01)	0.853 (0.009)	0.844 (0.016)	0.834 (0.02)
AVG50	0.858 (0.008)	0.859 (0.008)	0.857 (0.008)	0.859 (0.008)	0.856 (0.009)	0.848 (0.012)
AVG70	0.858 (0.008)	0.859 (0.008)	0.857 (0.008)	0.859 (0.008)	0.856 (0.009)	0.845 (0.012)
AVG90	0.859 (0.008)	0.859 (0.008)	0.858 (0.008)	0.859 (0.008)	0.854 (0.01)	0.841 (0.013)
STK2	0.858 (0.008)	0.859 (0.008)	0.857 (0.008)	0.858 (0.008)	0.857 (0.008)	0.858 (0.008)
GRP	0.857 (0.008)	0.859 (0.008)	0.857 (0.008)	0.859 (0.008)	0.852 (0.011)	0.852 (0.009)
Missing = 20%, MI = 10, Censoring = 0.30						
CC	0.84 (0.013)	0.842 (0.013)	0.839 (0.013)	0.842 (0.013)	0.807 (0.039)	0.794 (0.039)
AVG50	0.85 (0.01)	0.851 (0.01)	0.849 (0.01)	0.851 (0.01)	0.839 (0.016)	0.823 (0.017)
AVG70	0.851 (0.01)	0.851 (0.01)	0.849 (0.01)	0.851 (0.01)	0.836 (0.017)	0.821 (0.017)
AVG90	0.85 (0.011)	0.851 (0.01)	0.85 (0.01)	0.851 (0.011)	0.833 (0.017)	0.819 (0.016)
STK2	0.851 (0.01)	0.851 (0.01)	0.849 (0.01)	0.85 (0.01)	0.85 (0.01)	0.849 (0.011)
GRP	0.844 (0.013)	0.847 (0.014)	0.849 (0.01)	0.851 (0.01)	0.839 (0.014)	0.84 (0.014)

**Table 4:** Summary Statistic of variable selection by simulation settings for strong signal.

	Missing = 10%, MI = 10, Censoring = 0.10						Missing = 20%, MI = 10, Censoring = 0.10					
	CC	AVG50	AVG70	AVG90	STK2	GRP	CC	AVG50	AVG70	AVG90	STK2	GRP
Correctly Selection												
LASSO.BIC	0.00	0.00	0.00	0.14	0.00	0.02	0.00	0.01	0.05	0.28	0.02	0.00
ALASSO.BIC	0.75	0.76	0.92	0.98	0.80	0.92	0.66	0.70	0.87	0.97	0.77	0.69
LASSO.CV.min	0.00	0.00	0.02	0.28	0.00	0.08	0.00	0.00	0.03	0.30	0.00	0.02
ALASSO.CV.min	0.69	0.92	0.99	1.00	0.75	1.00	0.49	0.81	0.94	0.97	0.65	0.99
LASSO.CV.1se	0.05	0.67	0.82	0.93	0.00	0.15	0.00	0.62	0.81	0.92	0.00	0.09
ALASSO.CV.1se	1.00	1.00	1.00	1.00	0.95	1.00	1.00	1.00	0.99	0.97	0.90	1.00
Positively Discovery												
LASSO.BIC	0.54	0.57	0.69	0.86	0.67	0.67	0.55	0.60	0.74	0.88	0.70	0.63
ALASSO.BIC	0.97	0.97	0.99	1.00	0.97	0.99	0.96	0.96	0.99	1.00	0.97	0.96
LASSO.CV.min	0.50	0.61	0.74	0.90	0.50	0.74	0.51	0.61	0.74	0.89	0.50	0.66
ALASSO.CV.min	0.95	0.99	1.00	1.00	0.95	1.00	0.91	0.98	0.99	1.00	0.94	1.00
LASSO.CV.1se	0.76	0.95	0.98	0.99	0.66	0.84	0.77	0.95	0.98	0.99	0.67	0.81
ALASSO.CV.1se	1.00	1.00	1.00	1.00	1.00	1.00	1.00	1.00	1.00	1.00	0.98	1.00
False Positive												
LASSO.BIC	0.72	0.66	0.39	0.14	0.43	0.44	0.70	0.57	0.31	0.12	0.38	0.50
ALASSO.BIC	0.03	0.03	0.01	0.00	0.03	0.01	0.04	0.04	0.01	0.00	0.03	0.04
LASSO.CV.min	0.83	0.56	0.31	0.10	0.82	0.33	0.79	0.54	0.30	0.11	0.82	0.45
ALASSO.CV.min	0.06	0.01	0.00	0.00	0.06	0.00	0.09	0.02	0.01	0.00	0.07	0.00
LASSO.CV.1se	0.28	0.05	0.02	0.01	0.44	0.17	0.27	0.05	0.02	0.01	0.42	0.21
ALASSO.CV.1se	0.00	0.00	0.00	0.00	0.01	0.00	0.00	0.00	0.00	0.00	0.02	0.00
False Negative												
LASSO.BIC	0.00	0.00	0.00	0.00	0.00	0.00	0.00	0.00	0.00	0.00	0.00	0.00
ALASSO.BIC	0.00	0.00	0.00	0.00	0.00	0.00	0.00	0.00	0.00	0.00	0.00	0.00
LASSO.CV.min	0.00	0.00	0.00	0.00	0.00	0.00	0.00	0.00	0.00	0.00	0.00	0.00
ALASSO.CV.min	0.00	0.00	0.00	0.00	0.00	0.00	0.00	0.00	0.00	0.00	0.00	0.00
LASSO.CV.1se	0.00	0.00	0.00	0.00	0.00	0.00	0.00	0.00	0.00	0.00	0.00	0.00
ALASSO.CV.1se	0.00	0.00	0.00	0.00	0.00	0.00	0.00	0.00	0.00	0.00	0.00	0.00

**Table 5:** Summary Statistic of parameter estimates by simulation settings for strong signal.

	Missing = 10%, MI = 10, Censoring = 0.10						Missing = 20%, MI = 10, Censoring = 0.10					
	CC	AVG50	AVG70	AVG90	STK2	GRP	CC	AVG50	AVG70	AVG90	STK2	GRP
Bias												
LASSO.BIC	−0.27	−0.35	−0.35	−0.35	−0.36	−0.35	−0.27	−0.39	−0.39	−0.39	−0.40	−0.39
ALASSO.BIC	−0.28	−0.35	−0.35	−0.35	−0.36	−0.35	−0.28	−0.39	−0.39	−0.39	−0.40	−0.39
LASSO.CV.min	−0.27	−0.36	−0.36	−0.35	−0.36	−0.36	−0.27	−0.40	−0.39	−0.39	−0.40	−0.39
ALASSO.CV.min	−0.28	−0.36	−0.36	−0.36	−0.36	−0.36	−0.28	−0.39	−0.39	−0.39	−0.40	−0.39
LASSO.CV.1se	−0.34	−0.43	−0.43	−0.43	−0.39	−0.43	−0.35	−0.47	−0.47	−0.47	−0.43	−0.47
ALASSO.CV.1se	−0.34	−0.43	−0.43	−0.43	−0.38	−0.43	−0.35	−0.47	−0.47	−0.47	−0.42	−0.47
MSE												
LASSO.BIC	0.77	1.29	1.30	1.32	1.37	1.31	0.79	1.58	1.59	1.60	1.65	1.59
ALASSO.BIC	0.81	1.36	1.37	1.37	1.43	1.37	0.82	1.65	1.65	1.66	1.71	1.65
LASSO.CV.min	0.77	1.31	1.32	1.33	1.36	1.33	0.79	1.59	1.60	1.61	1.64	1.60
ALASSO.CV.min	0.80	1.38	1.38	1.38	1.42	1.38	0.82	1.66	1.66	1.67	1.70	1.66
LASSO.CV.1se	1.17	1.94	1.94	1.94	1.54	1.94	1.24	2.27	2.27	2.28	1.86	2.27
ALASSO.CV.1se	1.22	1.96	1.96	1.96	1.59	1.96	1.30	2.29	2.29	2.30	1.90	2.29

**Table 6:** Summary of tAUC (standard deviation) from ridge regression for the simulation with 10 multiple imputation using strong signal.

	LASSO.BIC	ALASSO.BIC	LASSO.CV.min	ALASSO.CV.min	LASSO.CV.1se	ALASSO.CV.1se
Missing = 10%, MI = 10, Censoring = 0.10						
CC	0.987 (0.002)	0.987 (0.002)	0.987 (0.002)	0.987 (0.002)	0.987 (0.002)	0.987 (0.002)
AVG50	0.986 (0.002)	0.986 (0.002)	0.986 (0.002)	0.986 (0.002)	0.986 (0.002)	0.986 (0.002)
AVG70	0.986 (0.002)	0.986 (0.002)	0.986 (0.002)	0.986 (0.002)	0.986 (0.002)	0.986 (0.002)
AVG90	0.986 (0.002)	0.986 (0.002)	0.986 (0.002)	0.986 (0.002)	0.987 (0.002)	0.986 (0.002)
STK2	0.985 (0.002)	0.986 (0.002)	0.985 (0.002)	0.986 (0.002)	0.985 (0.002)	0.986 (0.002)
GRP	0.986 (0.002)	0.986 (0.002)	0.986 (0.002)	0.986 (0.002)	0.986 (0.002)	0.986 (0.002)
Missing = 10%, MI = 10, Censoring = 0.30						
CC	0.984 (0.002)	0.984 (0.002)	0.984 (0.002)	0.984 (0.002)	0.984 (0.002)	0.984 (0.002)
AVG50	0.983 (0.002)	0.983 (0.002)	0.983 (0.002)	0.983 (0.002)	0.984 (0.002)	0.983 (0.002)
AVG70	0.983 (0.002)	0.983 (0.002)	0.983 (0.002)	0.983 (0.002)	0.984 (0.002)	0.983 (0.002)
AVG90	0.983 (0.002)	0.983 (0.002)	0.983 (0.002)	0.983 (0.002)	0.984 (0.002)	0.983 (0.003)
STK2	0.982 (0.002)	0.983 (0.002)	0.982 (0.002)	0.983 (0.002)	0.983 (0.002)	0.983 (0.002)
GRP	0.983 (0.002)	0.983 (0.002)	0.983 (0.002)	0.983 (0.002)	0.983 (0.002)	0.983 (0.002)
Missing = 20%, MI = 10, Censoring = 0.10						
CC	0.986 (0.002)	0.987 (0.002)	0.986 (0.002)	0.987 (0.002)	0.986 (0.002)	0.987 (0.002)
AVG50	0.984 (0.002)	0.985 (0.002)	0.984 (0.002)	0.985 (0.002)	0.985 (0.002)	0.985 (0.002)
AVG70	0.984 (0.002)	0.985 (0.002)	0.984 (0.002)	0.985 (0.002)	0.985 (0.002)	0.985 (0.003)
AVG90	0.984 (0.002)	0.985 (0.002)	0.984 (0.002)	0.985 (0.003)	0.985 (0.002)	0.985 (0.003)
STK2	0.983 (0.003)	0.984 (0.002)	0.983 (0.003)	0.984 (0.003)	0.984 (0.003)	0.984 (0.002)
GRP	0.984 (0.002)	0.985 (0.002)	0.984 (0.002)	0.985 (0.002)	0.985 (0.002)	0.985 (0.002)
Missing = 20%, MI = 10, Censoring = 0.30						
CC	0.983 (0.002)	0.984 (0.002)	0.983 (0.002)	0.984 (0.002)	0.983 (0.002)	0.984 (0.002)
AVG50	0.981 (0.002)	0.982 (0.002)	0.981 (0.002)	0.982 (0.002)	0.982 (0.002)	0.982 (0.003)
AVG70	0.981 (0.002)	0.982 (0.002)	0.981 (0.002)	0.982 (0.002)	0.983 (0.002)	0.982 (0.003)
AVG90	0.982 (0.002)	0.982 (0.002)	0.982 (0.002)	0.982 (0.002)	0.983 (0.003)	0.981 (0.004)
STK2	0.981 (0.003)	0.981 (0.003)	0.98 (0.003)	0.981 (0.003)	0.981 (0.003)	0.981 (0.003)
GRP	0.981 (0.002)	0.982 (0.002)	0.981 (0.002)	0.982 (0.002)	0.982 (0.002)	0.982 (0.003)

**Table 7:** Baseline characteristics of patients in CALGB 90401 study.

	Overall (N = 853)
Bone metastases	
No	238 (27.9%)
Yes	615 (72.1%)
Visceral metastases	
No	710 (83.2%)
Yes	143 (16.8%)
Opioid analgesic use	
No	435 (51.0%)
Yes	255 (29.9%)
Missing	163 (19.1%)
Age	
Median [Min, Max]	69.0 [42.0, 93.0]
BMI	
Median [Min, Max]	28.9 [15.0, 212]
Missing	106 (12.4%)
Race	
Other	101 (11.8%)
White	752 (88.2%)
ECOG Performance Status	
0	479 (56.2%)
1	341 (40.0%)
2	33 (3.9%)
Comorbidity	
0	265 (31.1%)
1	268 (31.4%)
2	161 (18.9%)
3	77 (9.0%)
4	47 (5.5%)
5	20 (2.3%)
6	6 (0.7%)
7	5 (0.6%)
8	2 (0.2%)
9	1 (0.1%)
Missing	1 (0.1%)
Gleason score	
2	1 (0.1%)
3	8 (0.9%)
4	8 (0.9%)
5	28 (3.3%)
6	94 (11.0%)
7	300 (35.2%)
8	156 (18.3%)
9	226 (26.5%)
10	30 (3.5%)
Missing	2 (0.2%)
Previous radiotherapy	
No	161 (18.9%)
Yes	692 (81.1%)
LDH >1 ULN	
No	536 (62.8%)
Yes	314 (36.8%)
Missing	3 (0.4%)
ALB	
Median [Min, Max]	4.00 [1.10, 5.70]
Missing	4 (0.5%)
BILI	
Median [Min, Max]	0.500 [0, 3.00]
HGB	
Median [Min, Max]	12.8 [6.60, 17.7]
PLT	
Median [Min, Max]	253 [15.0, 813]
Missing	1 (0.1%)
WBC	
Median [Min, Max]	6.30 [2.50, 17.6]
ALKPHOS *	
Median [Min, Max]	4.76 [3.53, 7.60]
AST	
Median [Min, Max]	25.0 [5.00, 161]
PSA *	
Median [Min, Max]	4.33 [−3.00, 9.21]

* Log transformed.

**Table 8:** Coefficient estimates for CALGB 90401 data.

	DS2	DS3	PAIN	ECOG	LDH.High	ALB	HGB	ALKPHOS	PSA	testo_m	Andro_m	deh_m
CC												
LASSO.BIC	0.25	0.37	0.26	0.30	0.38	−0.25	−0.10	0.00	0.00	0.00	−0.01	0.00
ALASSO.BIC	0.31	0.40	0.25	0.30	0.41	−0.27	−0.11	0.00	0.00	0.00	0.00	0.00
LASSO.CV.min	0.03	0.04	0.07	0.07	0.08	−0.07	−0.03	0.00	0.00	0.00	0.00	0.00
ALASSO.CV.min	0.31	0.40	0.25	0.30	0.41	−0.27	−0.11	0.00	0.00	0.00	0.00	0.00
LASSO.CV.1se	0.02	0.03	0.06	0.06	0.07	−0.06	−0.03	0.00	0.00	0.00	0.00	0.00
ALASSO.CV.1se	0.04	0.07	0.09	0.08	0.13	−0.08	−0.02	0.00	0.00	0.00	0.00	0.00
AVG50												
LASSO.BIC	0.13	0.32	0.12	0.26	0.28	−0.16	−0.10	0.15	0.07	0.00	0.00	0.00
ALASSO.BIC	0.16	0.35	0.09	0.25	0.29	−0.10	−0.09	0.15	0.06	0.00	0.00	0.00
LASSO.CV.min	0.05	0.19	0.09	0.19	0.22	−0.10	−0.07	0.13	0.05	0.00	−0.03	0.00
ALASSO.CV.min	0.11	0.29	0.08	0.24	0.29	−0.10	−0.07	0.13	0.04	0.00	0.00	0.00
LASSO.CV.1se	0.02	0.08	0.08	0.10	0.13	−0.09	−0.04	0.09	0.03	0.00	0.00	0.00
ALASSO.CV.1se	0.03	0.12	0.05	0.13	0.17	−0.08	−0.03	0.07	0.01	0.00	0.00	0.00
AVG70												
LASSO.BIC	0.13	0.32	0.12	0.26	0.28	−0.16	−0.10	0.15	0.07	0.00	0.00	0.00
ALASSO.BIC	0.16	0.36	0.12	0.28	0.30	−0.17	−0.09	0.15	0.07	0.00	0.00	0.00
LASSO.CV.min	0.07	0.22	0.10	0.20	0.24	−0.11	−0.08	0.14	0.05	0.00	0.00	0.00
ALASSO.CV.min	0.11	0.29	0.08	0.24	0.29	−0.10	−0.07	0.13	0.04	0.00	0.00	0.00
LASSO.CV.1se	0.01	0.06	0.07	0.08	0.11	−0.08	−0.04	0.08	0.03	0.00	0.00	0.00
ALASSO.CV.1se	0.03	0.12	0.05	0.14	0.18	−0.08	−0.03	0.07	0.02	0.00	0.00	0.00
AVG90												
LASSO.BIC	0.13	0.32	0.12	0.26	0.28	−0.16	−0.10	0.15	0.07	0.00	0.00	0.00
ALASSO.BIC	0.16	0.36	0.12	0.28	0.30	−0.17	−0.09	0.15	0.07	0.00	0.00	0.00
LASSO.CV.min	0.08	0.25	0.11	0.23	0.26	−0.14	−0.09	0.14	0.06	0.00	0.00	0.00
ALASSO.CV.min	0.13	0.31	0.11	0.27	0.30	−0.16	−0.08	0.15	0.05	0.00	0.00	0.00
LASSO.CV.1se	0.01	0.06	0.06	0.08	0.10	−0.07	−0.03	0.07	0.02	0.00	0.00	0.00
ALASSO.CV.1se	0.03	0.12	0.05	0.13	0.17	−0.08	−0.02	0.07	0.01	0.00	0.00	0.00
STK2												
LASSO.BIC	0.13	0.32	0.12	0.26	0.28	−0.16	−0.10	0.15	0.07	0.00	0.00	0.00
ALASSO.BIC	0.16	0.36	0.12	0.28	0.30	−0.17	−0.09	0.15	0.07	0.00	0.00	0.00
LASSO.CV.min	0.14	0.32	0.08	0.24	0.25	−0.09	−0.08	0.10	0.06	−0.01	−0.05	0.00
ALASSO.CV.min	0.18	0.38	0.07	0.26	0.27	−0.08	−0.07	0.10	0.05	0.00	−0.05	0.00
LASSO.CV.1se	0.04	0.15	0.09	0.16	0.19	−0.10	−0.06	0.12	0.04	0.00	−0.02	0.00
ALASSO.CV.1se	0.07	0.22	0.07	0.21	0.26	−0.08	−0.05	0.11	0.03	0.00	−0.01	0.00
GRP												
LASSO.BIC	0.00	0.23	0.00	0.29	0.28	0.00	−0.11	0.15	0.07	0.00	0.00	0.00
ALASSO.BIC	0.00	0.20	0.00	0.38	0.47	0.00	0.00	0.00	0.00	0.00	0.00	0.00
LASSO.CV.min	0.00	0.24	0.00	0.27	0.00	0.00	−0.12	0.22	0.08	0.00	0.00	0.00
ALASSO.CV.min	0.00	0.00	0.00	0.00	0.00	0.00	0.00	0.00	0.00	0.00	0.00	0.00
LASSO.CV.1se	0.00	0.00	0.00	0.10	0.00	0.00	−0.05	0.10	0.00	0.00	0.00	0.00
ALASSO.CV.1se	0.00	0.00	0.00	0.00	0.00	0.00	0.00	0.00	0.00	0.00	0.00	0.00
	ANG2	BMP9	CD73	ChromograninA	HER3	HGF	ICAM1	IL6	OPN	PDGFAA	PDGFbb	PlGF
CC												
LASSO.BIC	0.00	0.00	0.00	0.00	0.00	0.00	0.00	0.00	0.00	0.00	0.00	0.00
ALASSO.BIC	0.00	0.00	0.00	0.00	0.00	0.00	0.00	0.00	0.00	0.00	0.00	0.00
LASSO.CV.min	0.00	0.00	0.00	0.00	0.00	0.00	0.00	0.00	0.00	0.00	0.00	0.00
ALASSO.CV.min	0.00	0.00	0.00	0.00	0.00	0.00	0.00	0.00	0.00	0.00	0.00	0.00
LASSO.CV.1se	0.00	0.00	0.00	0.00	0.00	0.00	0.00	0.00	0.00	0.00	0.00	0.00
ALASSO.CV.1se	0.00	0.00	0.00	0.00	0.00	0.00	0.00	0.00	0.00	0.00	0.00	0.00
AVG50												
LASSO.BIC	0.00	0.00	0.00	0.00	0.00	0.00	0.00	0.00	0.00	0.00	0.00	0.00
ALASSO.BIC	0.00	0.00	0.00	0.00	0.00	0.00	0.28	0.00	0.00	0.00	0.00	0.00
LASSO.CV.min	0.08	0.00	0.00	0.00	0.00	0.00	0.17	0.00	0.00	0.00	0.00	0.00
ALASSO.CV.min	0.00	0.00	0.00	0.00	0.00	0.00	0.23	0.00	0.00	0.00	0.00	0.00
LASSO.CV.1se	0.00	0.00	0.00	0.00	0.00	0.00	0.00	0.00	0.00	0.00	0.00	0.00
ALASSO.CV.1se	0.00	0.00	0.00	0.00	0.00	0.00	0.00	0.00	0.00	0.00	0.00	0.00
AVG70												
LASSO.BIC	0.00	0.00	0.00	0.00	0.00	0.00	0.00	0.00	0.00	0.00	0.00	0.00
ALASSO.BIC	0.00	0.00	0.00	0.00	0.00	0.00	0.00	0.00	0.00	0.00	0.00	0.00
LASSO.CV.min	0.00	0.00	0.00	0.00	0.00	0.00	0.20	0.00	0.00	0.00	0.00	0.00
ALASSO.CV.min	0.00	0.00	0.00	0.00	0.00	0.00	0.23	0.00	0.00	0.00	0.00	0.00
LASSO.CV.1se	0.00	0.00	0.00	0.00	0.00	0.00	0.00	0.00	0.00	0.00	0.00	0.00
ALASSO.CV.1se	0.00	0.00	0.00	0.00	0.00	0.00	0.00	0.00	0.00	0.00	0.00	0.00
AVG90												
LASSO.BIC	0.00	0.00	0.00	0.00	0.00	0.00	0.00	0.00	0.00	0.00	0.00	0.00
ALASSO.BIC	0.00	0.00	0.00	0.00	0.00	0.00	0.00	0.00	0.00	0.00	0.00	0.00
LASSO.CV.min	0.00	0.00	0.00	0.00	0.00	0.00	0.29	0.00	0.00	0.00	0.00	0.00
ALASSO.CV.min	0.00	0.00	0.00	0.00	0.00	0.00	0.00	0.00	0.00	0.00	0.00	0.00
LASSO.CV.1se	0.00	0.00	0.00	0.00	0.00	0.00	0.00	0.00	0.00	0.00	0.00	0.00
ALASSO.CV.1se	0.00	0.00	0.00	0.00	0.00	0.00	0.00	0.00	0.00	0.00	0.00	0.00
STK2												
LASSO.BIC	0.00	0.00	0.00	0.00	0.00	0.00	0.00	0.00	0.00	0.00	0.00	0.00
ALASSO.BIC	0.00	0.00	0.00	0.00	0.00	0.00	0.00	0.00	0.00	0.00	0.00	0.00
LASSO.CV.min	0.05	−0.04	−0.03	0.02	0.02	0.05	0.17	−0.02	0.03	−0.07	0.02	0.04
ALASSO.CV.min	0.04	−0.04	−0.02	0.01	0.00	0.04	0.18	−0.01	0.02	−0.07	0.01	0.04
LASSO.CV.1se	0.06	−0.04	0.00	0.00	0.00	0.03	0.14	0.00	0.00	0.00	0.00	0.05
ALASSO.CV.1se	0.00	0.00	0.00	0.00	0.00	0.00	0.18	0.00	0.00	−0.03	0.00	0.00
GRP												
LASSO.BIC	0.00	0.00	0.00	0.00	0.00	0.00	0.36	0.00	0.00	0.00	0.00	0.00
ALASSO.BIC	0.00	0.00	0.00	0.00	0.00	0.00	0.38	0.00	0.00	0.00	0.00	0.00
LASSO.CV.min	0.00	0.00	0.00	0.00	0.00	0.00	0.00	0.00	0.00	0.00	0.00	0.00
ALASSO.CV.min	0.00	0.00	0.00	0.00	0.00	0.00	0.00	0.00	0.00	0.00	0.00	0.00
LASSO.CV.1se	0.00	0.00	0.00	0.00	0.00	0.00	0.00	0.00	0.00	0.00	0.00	0.00
ALASSO.CV.1se	0.00	0.00	0.00	0.00	0.00	0.00	0.00	0.00	0.00	0.00	0.00	0.00
	SDF1	TGFb1	TGFb2	TGFbR3	TIMP	TSP2	VCAM1	VEGFA	VEGFD	VEGFR1	VEGFR2	VEGFR3
CC												
LASSO.BIC	0.00	0.00	0.00	0.00	0.00	0.00	0.00	0.00	0.00	0.00	0.00	0.00
ALASSO.BIC	0.00	0.00	0.00	0.00	0.00	0.00	0.00	0.00	0.00	0.00	0.00	0.00
LASSO.CV.min	0.00	0.00	0.00	0.00	0.00	0.00	0.00	0.00	0.00	0.00	0.00	0.00
ALASSO.CV.min	0.00	0.00	0.00	0.00	0.00	0.00	0.00	0.00	0.00	0.00	0.00	0.00
LASSO.CV.1se	0.00	0.00	0.00	0.00	0.00	0.00	0.00	0.00	0.00	0.00	0.00	0.00
ALASSO.CV.1se	0.00	0.00	0.00	0.00	0.00	0.00	0.00	0.00	0.00	0.00	0.00	0.00
AVG50												
LASSO.BIC	0.00	0.00	0.00	0.00	0.00	0.00	0.00	0.00	0.00	0.00	0.00	0.00
ALASSO.BIC	0.00	0.00	0.00	0.00	0.16	0.00	0.00	0.00	0.00	0.00	0.00	0.00
LASSO.CV.min	0.00	0.00	0.00	0.00	0.10	0.00	0.00	0.04	0.00	0.00	0.00	0.14
ALASSO.CV.min	0.00	0.00	0.00	0.00	0.12	0.00	0.00	0.00	0.00	0.00	0.00	0.19
LASSO.CV.1se	0.00	0.00	0.00	0.00	0.00	0.00	0.00	0.00	0.00	0.00	0.00	0.00
ALASSO.CV.1se	0.00	0.00	0.00	0.00	0.00	0.00	0.00	0.00	0.00	0.00	0.00	0.00
AVG70												
LASSO.BIC	0.00	0.00	0.00	0.00	0.00	0.00	0.00	0.00	0.00	0.00	0.00	0.00
ALASSO.BIC	0.00	0.00	0.00	0.00	0.00	0.00	0.00	0.00	0.00	0.00	0.00	0.00
LASSO.CV.min	0.00	0.00	0.00	0.00	0.10	0.00	0.00	0.05	0.00	0.00	0.00	0.17
ALASSO.CV.min	0.00	0.00	0.00	0.00	0.12	0.00	0.00	0.00	0.00	0.00	0.00	0.19
LASSO.CV.1se	0.00	0.00	0.00	0.00	0.00	0.00	0.00	0.00	0.00	0.00	0.00	0.00
ALASSO.CV.1se	0.00	0.00	0.00	0.00	0.00	0.00	0.00	0.00	0.00	0.00	0.00	0.00
AVG90												
LASSO.BIC	0.00	0.00	0.00	0.00	0.00	0.00	0.00	0.00	0.00	0.00	0.00	0.00
ALASSO.BIC	0.00	0.00	0.00	0.00	0.00	0.00	0.00	0.00	0.00	0.00	0.00	0.00
LASSO.CV.min	0.00	0.00	0.00	0.00	0.00	0.00	0.00	0.00	0.00	0.00	0.00	0.00
ALASSO.CV.min	0.00	0.00	0.00	0.00	0.00	0.00	0.00	0.00	0.00	0.00	0.00	0.00
LASSO.CV.1se	0.00	0.00	0.00	0.00	0.00	0.00	0.00	0.00	0.00	0.00	0.00	0.00
ALASSO.CV.1se	0.00	0.00	0.00	0.00	0.00	0.00	0.00	0.00	0.00	0.00	0.00	0.00
STK2												
LASSO.BIC	0.00	0.00	0.00	0.00	0.00	0.00	0.00	0.00	0.00	0.00	0.00	0.00
ALASSO.BIC	0.00	0.00	0.00	0.00	0.00	0.00	0.00	0.00	0.00	0.00	0.00	0.00
LASSO.CV.min	0.02	−0.03	−0.07	−0.05	0.18	0.04	0.02	0.08	−0.02	−0.02	0.09	0.14
ALASSO.CV.min	0.01	−0.02	−0.07	−0.04	0.19	0.03	0.00	0.08	−0.01	−0.01	0.09	0.14
LASSO.CV.1se	0.00	0.00	−0.05	0.00	0.10	0.00	0.00	0.04	0.00	0.00	0.05	0.12
ALASSO.CV.1se	0.00	0.00	−0.02	0.00	0.13	0.00	0.00	0.03	0.00	0.00	0.03	0.14
GRP												
LASSO.BIC	0.00	0.00	0.00	0.00	0.00	0.00	0.00	0.00	0.00	0.00	0.00	0.00
ALASSO.BIC	0.00	0.00	0.00	0.00	0.00	0.00	0.00	0.00	0.00	0.00	0.00	0.30
LASSO.CV.min	0.00	0.00	0.00	0.00	0.00	0.00	0.00	0.00	0.00	0.00	0.00	0.00
ALASSO.CV.min	0.00	0.00	0.00	0.00	0.00	0.00	0.00	0.00	0.00	0.00	0.00	0.00
LASSO.CV.1se	0.00	0.00	0.00	0.00	0.00	0.00	0.00	0.00	0.00	0.00	0.00	0.00
ALASSO.CV.1se	0.00	0.00	0.00	0.00	0.00	0.00	0.00	0.00	0.00	0.00	0.00	0.00

**Table 9:** Optimism-corrected tAUCs (standard deviation) for CALGB 90401 data.

	LASSO.BIC	ALASSO.BIC	LASSO.CV.min	ALASSO.CV.min	LASSO.CV.1se	ALASSO.CV.1se
CC	0.7418 (0.0198)	0.7413 (0.0199)	0.7441 (0.0206)	0.7411 (0.0198)	0.7476 (0.0205)	0.7389 (0.0199)
AVG50	0.7349 (0.0172)	0.7339 (0.0167)	0.7369 (0.0174)	0.7344 (0.0169)	0.7374 (0.0174)	0.7322 (0.0168)
AVG70	0.7328 (0.0166)	0.7321 (0.0165)	0.7339 (0.0166)	0.7323 (0.0162)	0.7353 (0.0169)	0.7301 (0.0162)
AVG90	0.7296 (0.0156)	0.7288 (0.0155)	0.7301 (0.0156)	0.7285 (0.0155)	0.732 (0.0159)	0.7269 (0.0151)
STK2	0.7401 (0.0161)	0.7393 (0.016)	0.7343 (0.0162)	0.7357 (0.0161)	0.7432 (0.0166)	0.7388 (0.0157)
GRP	0.7239 (0.0205)	0.7233 (0.0213)	0.7265 (0.0206)	NA	0.7271 (0.0217)	NA

Note: “NA” indicates the model selected no variables; therefore, tAUC was not available.

**Table 10:** Integrated Brier Score for CALGB 90401 data.

	LASSO.BIC	ALASSO.BIC	LASSO.CV.min	ALASSO.CV.min	LASSO.CV.1se	ALASSO.CV.1se
CC	0.125	0.125	0.126	0.125	0.126	0.126
AVG50	0.123	0.122	0.121	0.121	0.124	0.124
AVG70	0.123	0.123	0.121	0.121	0.124	0.124
AVG90	0.123	0.123	0.122	0.123	0.124	0.124
STK2	0.123	0.123	0.119	0.119	0.119	0.120
GRP	0.123	0.127	0.126	NA	0.127	NA

Note: “NA” indicates the model selected no variables; therefore, integrated Brier score was not available.

**Table 11:** Calibration slope for CALGB 90401 data.

	LASSO.BIC	ALASSO.BIC	LASSO.CV.min	ALASSO.CV.min	LASSO.CV.1se	ALASSO.CV.1se
CC	0.983	0.932	3.888	0.932	11.470	2.761
AVG50	1.051	1.048	1.187	1.145	3.195	3.153
AVG70	1.051	1.049	1.156	1.140	4.139	3.375
AVG90	1.051	1.049	1.122	1.124	3.497	3.445
STK2	1.052	1.049	1.042	1.037	1.259	1.362
GRP	1.057	1.061	1.096	NA	4.974	NA

Note: “NA” indicates the model selected no variables; therefore, calibration slope was not available.

## Data Availability

The datasets used in the application section are publicly available via NCTN NCORP Data Archive. All the programs for simulation and application were written in R version 4.2.1 and are available upon reasonable request from the corresponding author.

## Supplementary Materials

The following supporting information can be downloaded at https://www.mdpi.com/article/10.3390/bioengineering12111278/s1; Table S1. Mean CI coverage of the true covariates; Table S2. Complete-case Cox proportional hazards model estimates for baseline clinical and biomarker variables in CALGB 90401; Figure S1. Variable selection—correctly selected predictors for weak signal scenarios across MI levels, missingness (10%, 20%), and censoring (0.10, 0.30); Figure S2. Variable selection—positive discovery/selection rate for weak signal scenarios across MI levels, missingness (10%, 20%), and censoring (0.10, 0.30); Figure S3. Variable selection—false positives for weak signal scenarios across MI levels, missingness (10%, 20%), and censoring (0.10, 0.30); Figure S4. Variable selection—false negatives for weak signal scenarios across MI levels, missingness (10%, 20%), and censoring (0.10, 0.30); Figure S5. Parameter estimation—average bias across methods and tuning settings for weak signal scenarios across MI levels, missingness (10%, 20%), and censoring (0.10, 0.30); Figure S6. Parameter estimation—average MSE across methods and tuning settings for weak signal scenarios across MI levels, missingness (10%, 20%), and censoring (0.10, 0.30); Figure S7. tAUC (mean and SD) from ridge refits for weak signal scenarios across MI levels, missingness (10%, 20%), and censoring (0.10, 0.30); Figure S8. Integrated Brier Score (mean and SD) for weak signal scenarios across MI levels, missingness (10%, 20%), and censoring (0.10, 0.30); Figure S9. Calibration slope (mean and SD) for weak signal scenarios across MI levels, missingness (10%, 20%), and censoring (0.10, 0.30); Figure S10. Calibration intercept at the 25th percentile (mean and SD) for weak signal scenarios across MI levels, missingness (10%, 20%), and censoring (0.10, 0.30); Figure S11. Calibration intercept at the 50th percentile (mean and SD) for weak signal scenarios across MI levels, missingness (10%, 20%), and censoring (0.10, 0.30); Figure S12. Calibration intercept at the 75th percentile (mean and SD) for weak signal scenarios across MI levels, missingness (10%, 20%), and censoring (0.10, 0.30); Figure S13. Variable selection—correctly selected predictors for strong signal scenarios across MI levels, missingness (10%, 20%), and censoring (0.10, 0.30); Figure S14. Variable selection—positive discovery/selection rate for strong signal scenarios across MI levels, missingness (10%, 20%), and censoring (0.10, 0.30); Figure S15. Variable selection—false positives for strong signal scenarios across MI levels, missingness (10%, 20%), and censoring (0.10, 0.30); Figure S16. Variable selection—false negatives for strong signal scenarios across MI levels, missingness (10%, 20%), and censoring (0.10, 0.30); Figure S17. Parameter estimation—average bias across methods and tuning settings for strong signal scenarios across MI levels, missingness (10%, 20%), and censoring (0.10, 0.30); Figure S18. Parameter estimation—average MSE across methods and tuning settings for strong signal scenarios across MI levels, missingness (10%, 20%), and censoring (0.10, 0.30); Figure S19. tAUC (mean and SD) from ridge refits for strong signal scenarios across MI levels, missingness (10%, 20%), and censoring (0.10, 0.30); Figure S20. Integrated Brier Score (mean and SD) for strong signal scenarios across MI levels, missingness (10%, 20%), and censoring (0.10, 0.30); Figure S21. Calibration slope (mean and SD) for strong signal scenarios across MI levels, missingness (10%, 20%), and censoring (0.10, 0.30); Figure S22. Calibration intercept at the 25th percentile (mean and SD) for strong signal scenarios across MI levels, missingness (10%, 20%), and censoring (0.10, 0.30); Figure S23. Calibration intercept at the 50th percentile (mean and SD) for strong signal scenarios across MI levels, missingness (10%, 20%), and censoring (0.10, 0.30); Figure S24. Calibration intercept at the 75th percentile (mean and SD) for strong signal scenarios across MI levels, missingness (10%, 20%), and censoring (0.10, 0.30); Figure S25. Kaplan–Meier curve of overall survival in the CALGB 90401 data.

## Abbreviations

The following abbreviations are used in this manuscript:

AIC	Akaike’s information criteria
ALASSO	Adaptive least absolute shrinkage and selection operator
BIC	Bayesian information criteria
CC	Complete cases
HR	Hazard ratio
IBS	Integrated Brier Score
LASSO	Least absolute shrinkage and selection operator
MAR	Missing at random
MCAR	Missing completely at random
mCRPC	Metastatic castration-resistant prostate cancer
MI	Multiple imputation
MNAR	Missing not at random
OS	Overall survival
tAUC	Time-dependent area under the curve
