# Supplementary material for: A Two-Step Variable Selection Strategy for Multiply Imputed Survival Data Using Penalized Cox Models
Source: Bioengineering (Basel). 2025 Nov 20;12(11):1278. doi: 10.3390/bioengineering12111278 (PMC12650151; doi:10.3390/bioengineering12111278)
Supplement: Supplementary file 1 [file bioengineering-12-01278-s001.zip › bioengineering-3877835-supplementary.pdf]

Supplementary Materials

Supplemental Table S1. Mean CI coverage of the true covariates.

	Mean CI Coverage		
Covariate	AVG50	STK2	GRP
X1	0.74	0.20	0.71
X2	0.57	0.12	0.52
X3	0.75	0.26	0.70
X4	0.22	0.00	0.21
X5	0.80	0.31	0.77
X6	0.77	0.21	0.72
X7	0.78	0.34	0.60
X8	0.77	0.22	0.73
X9	0.63	0.09	0.60

Supplemental Table S2. Complete-case Cox proportional hazards model estimates for baseline clinical and biomarker variables in CALGB 90401.

Covariate	HR	95% CI Lower	95% CI Upper	P Value
DS2	1.3987	1.0195	1.9189	0.038
DS3	1.4931	1.0245	2.1760	0.037
PAIN	1.2658	1.0255	1.5623	0.028
ECOG	1.3837	1.1441	1.6735	0.001
LDH.High	1.5206	1.2215	1.8930	0.000
ALB	0.8595	0.6892	1.0719	0.179
HGB	0.9100	0.8492	0.9750	0.007
ALKPHOS	0.9999	0.9994	1.0004	0.727
PSA	1.0005	1.0002	1.0007	0.000
testo_m	1.0024	0.9978	1.0070	0.310
Andro_m	0.9905	0.9850	0.9960	0.001
deh_m	1.0022	0.9998	1.0045	0.067
ANG2	1.0001	0.9998	1.0004	0.476
BMP9	0.9997	0.9992	1.0003	0.334
CD73	1.0000	1.0000	1.0000	0.846
ChromograninA	1.0000	1.0000	1.0000	0.357
HER3	1.0000	1.0000	1.0000	0.491
HGF	1.0000	0.9999	1.0001	0.630
ICAM1	1.0000	1.0000	1.0000	0.244
IL6	0.9994	0.9988	1.0000	0.069
OPN	1.0000	1.0000	1.0000	0.386
PDGFAA	1.0000	0.9999	1.0001	0.659
PDGFbb	1.0000	1.0000	1.0001	0.330
PlGF	1.0030	0.9989	1.0072	0.147
SDF1	1.0000	1.0000	1.0001	0.206

TGFb1	1.0000	1.0000	1.0000	0.198
TGFb2	0.9991	0.9980	1.0001	0.066
TGFbR3	1.0000	1.0000	1.0000	0.720
TIMP	1.0000	1.0000	1.0000	0.014
TSP2	1.0000	1.0000	1.0000	0.574
VCAM1	1.0000	1.0000	1.0000	0.920
VEGFA	1.0003	1.0000	1.0007	0.075
VEGFD	1.0000	0.9999	1.0000	0.265
VEGFR1	1.0000	0.9997	1.0002	0.856
VEGFR2	1.0000	1.0000	1.0001	0.979
VEGFR3	1.0000	1.0000	1.0000	0.006

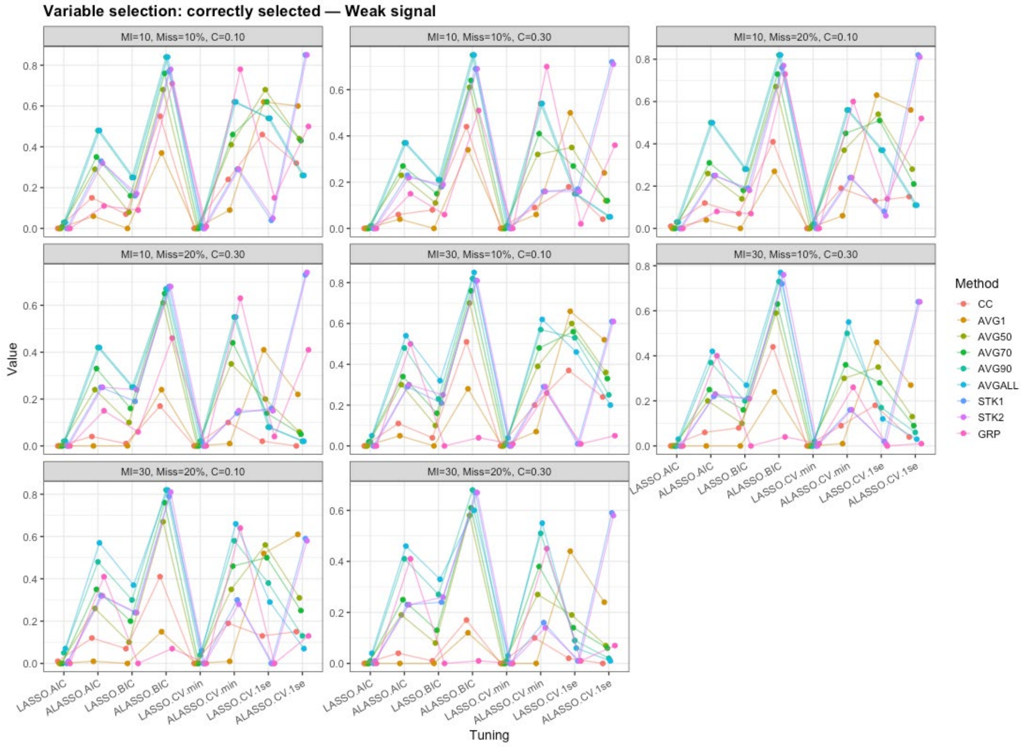

Supplemental Figure S1. Variable selection — correctly selected predictors for **weak** signal scenarios across MI levels, missingness (10%, 20%), and censoring (0.10, 0.30).

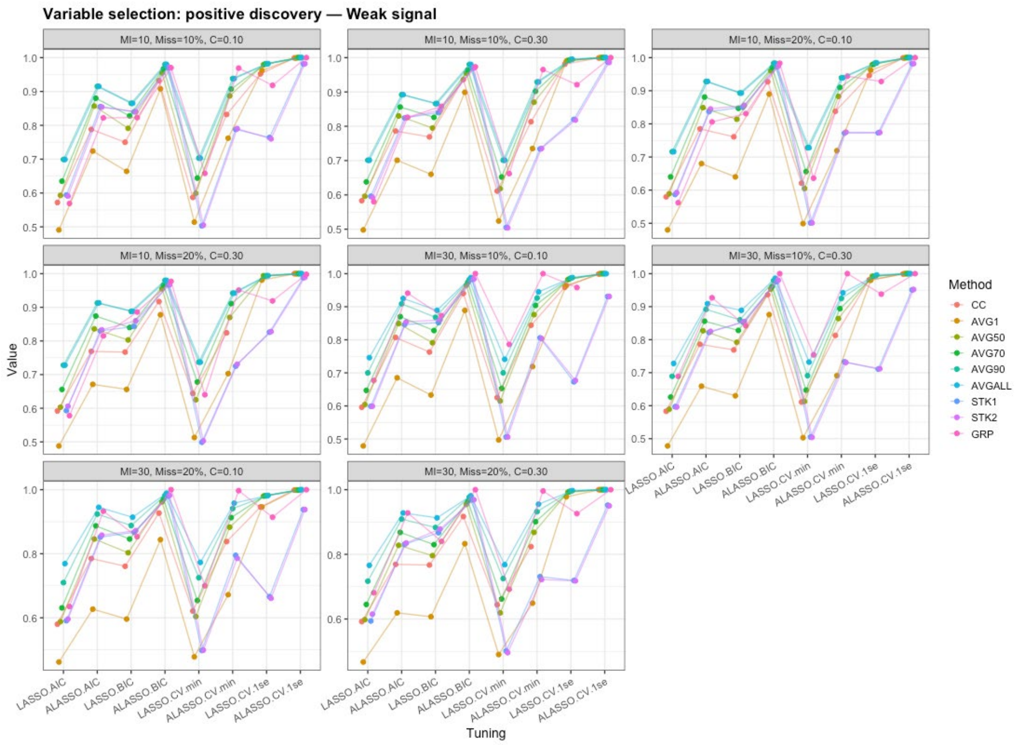

Supplemental Figure S2. Variable selection—positive discovery / selection rate for **weak** signal scenarios across MI levels, missingness (10%, 20%), and censoring (0.10, 0.30).

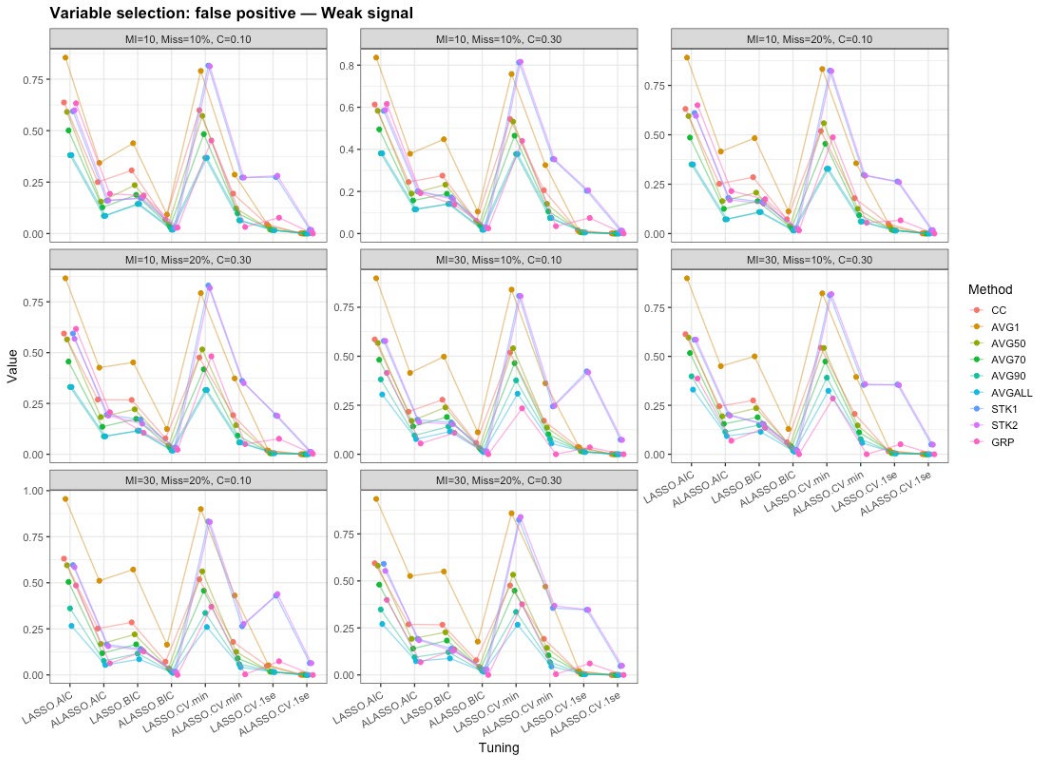

Supplemental Figure S3. Variable selection—false positives for **weak** signal scenarios across MI levels, missingness (10%, 20%), and censoring (0.10, 0.30).

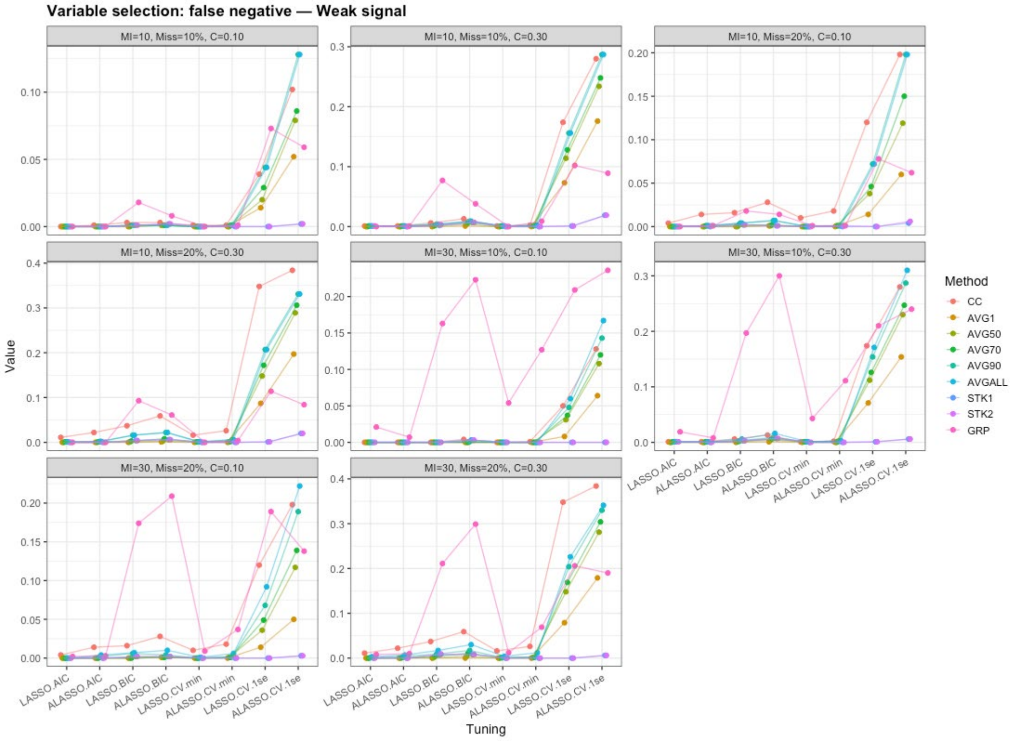

Supplemental Figure S4. Variable selection—false negatives for **weak** signal scenarios across MI levels, missingness (10%, 20%), and censoring (0.10, 0.30).

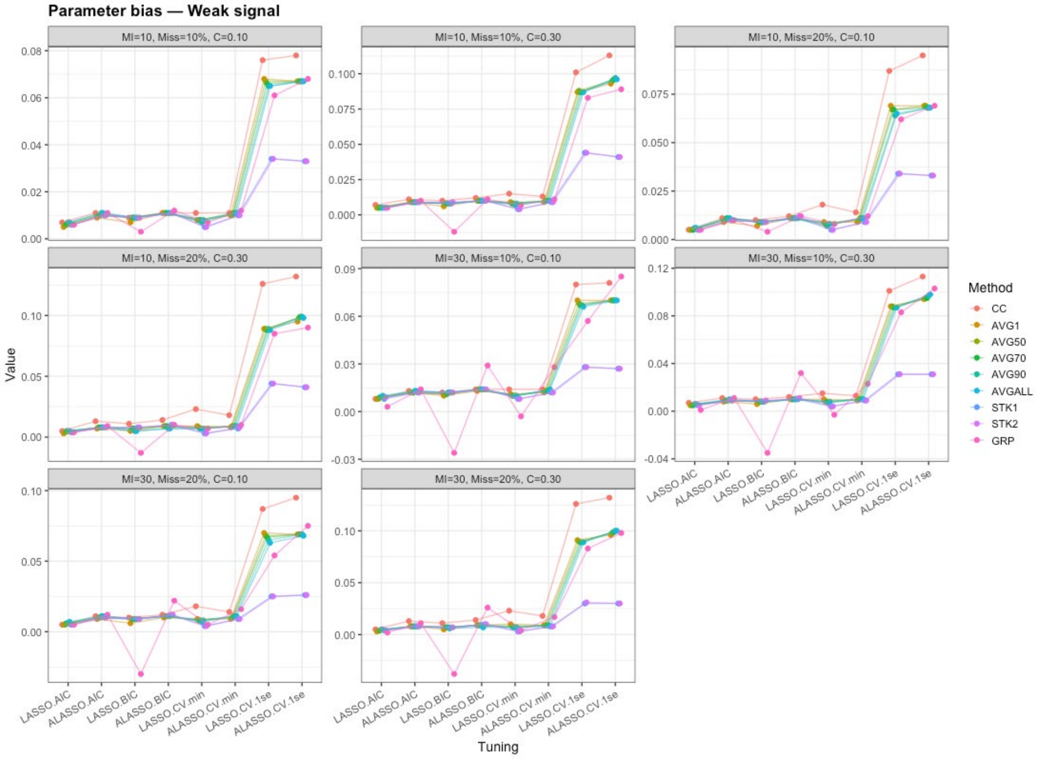

Supplemental Figure S5. Parameter estimation—average bias across methods and tuning settings for **weak** signal scenarios across MI levels, missingness (10%, 20%), and censoring (0.10, 0.30).

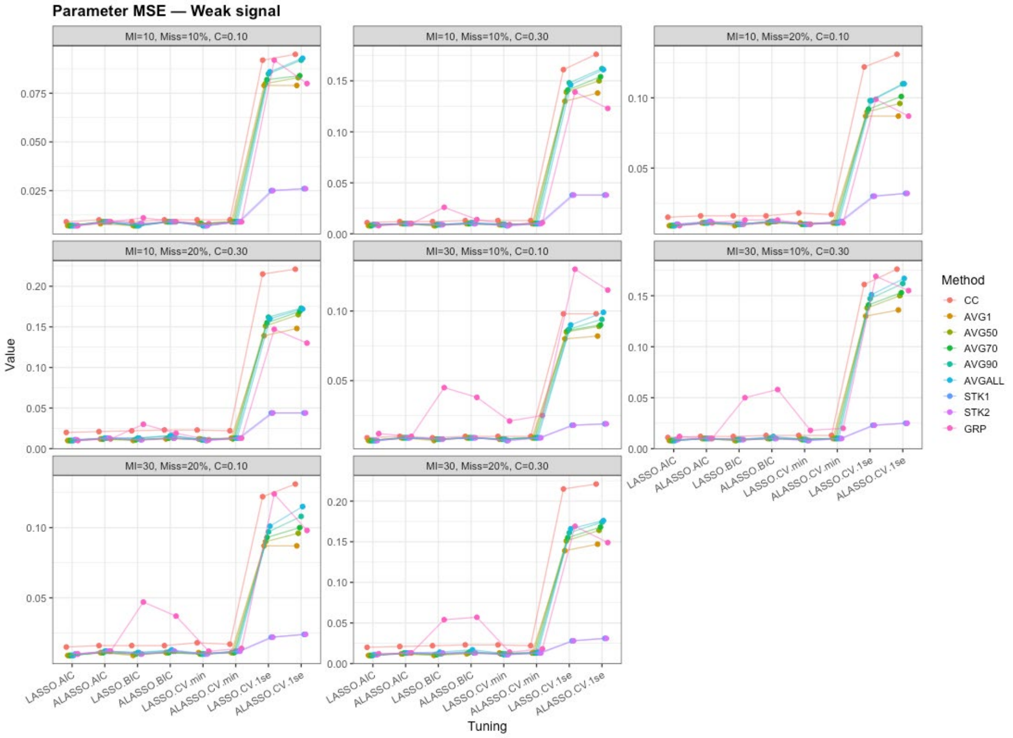

Supplemental Figure S6. Parameter estimation—average MSE across methods and tuning settings for **weak** signal scenarios across MI levels, missingness (10%, 20%), and censoring (0.10, 0.30).

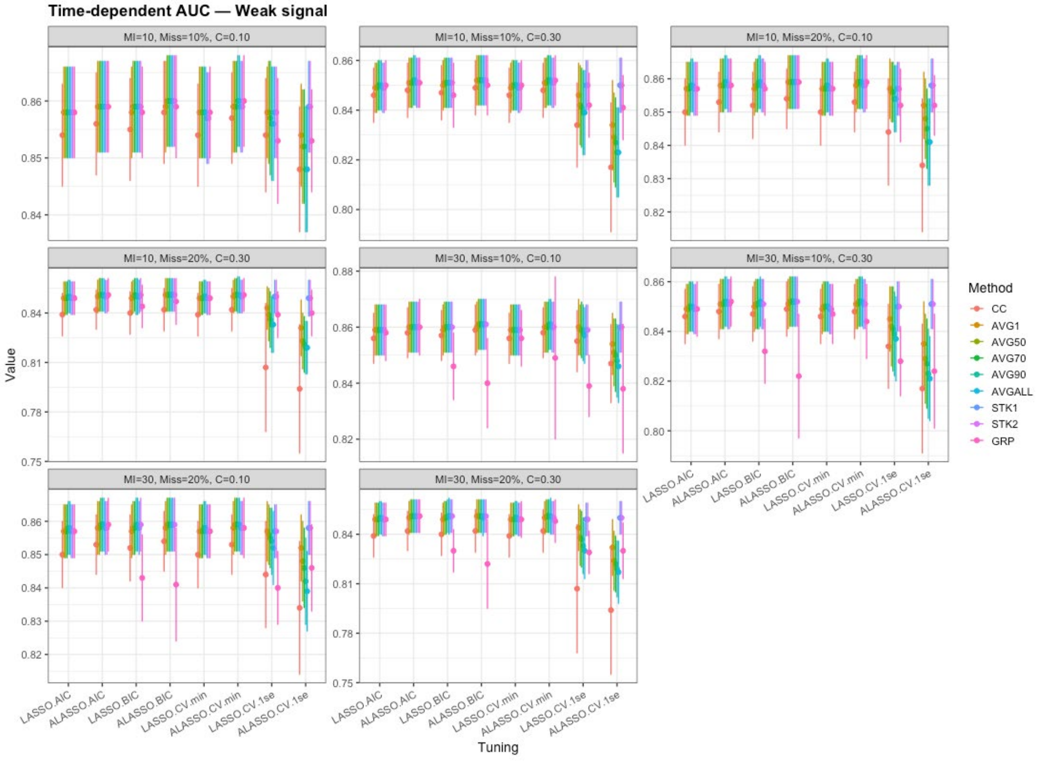

Supplemental Figure S7. tAUC (mean and SD) from ridge refits for **weak** signal scenarios across MI levels, missingness (10%, 20%), and censoring (0.10, 0.30).

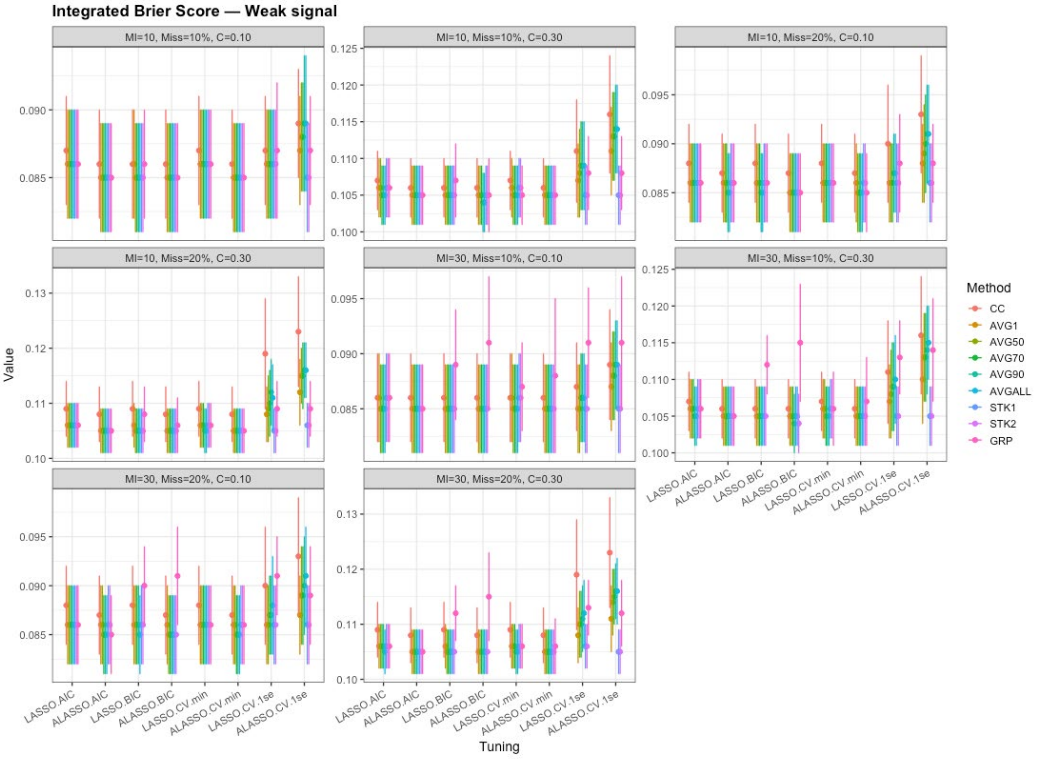

Supplemental Figure S8. Integrated Brier Score (mean and SD) for **weak** signal scenarios across MI levels, missingness (10%, 20%), and censoring (0.10, 0.30).

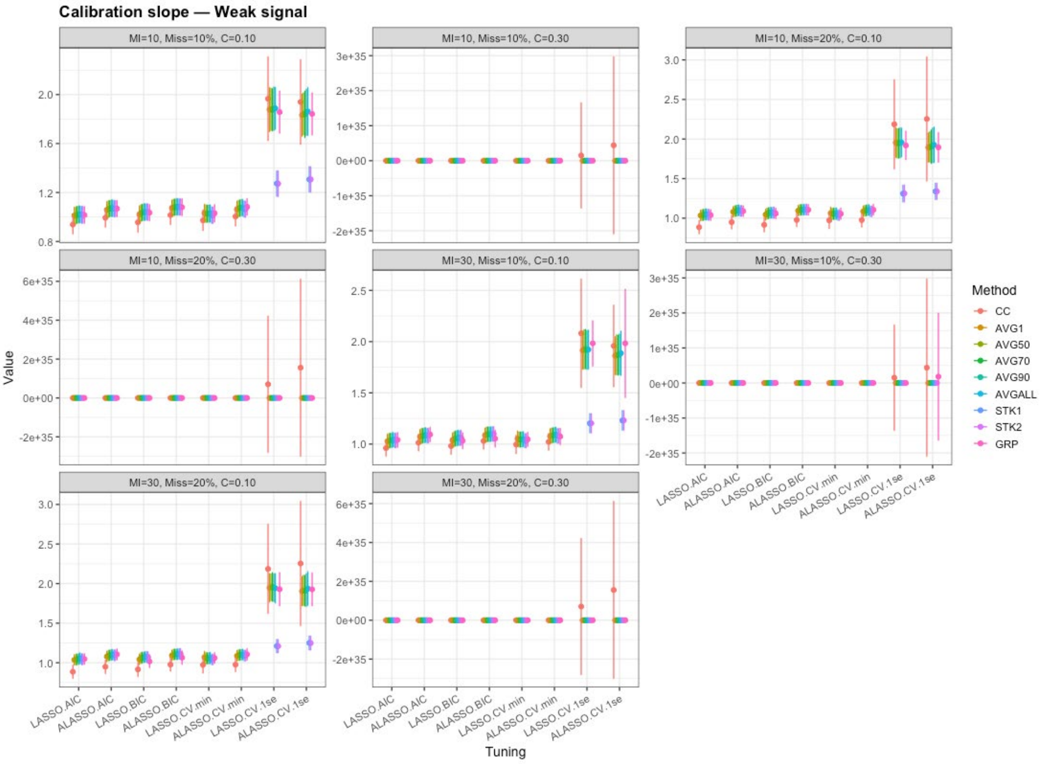

Supplemental Figure S9. Calibration slope (mean and SD) for **weak** signal scenarios across MI levels, missingness (10%, 20%), and censoring (0.10, 0.30).

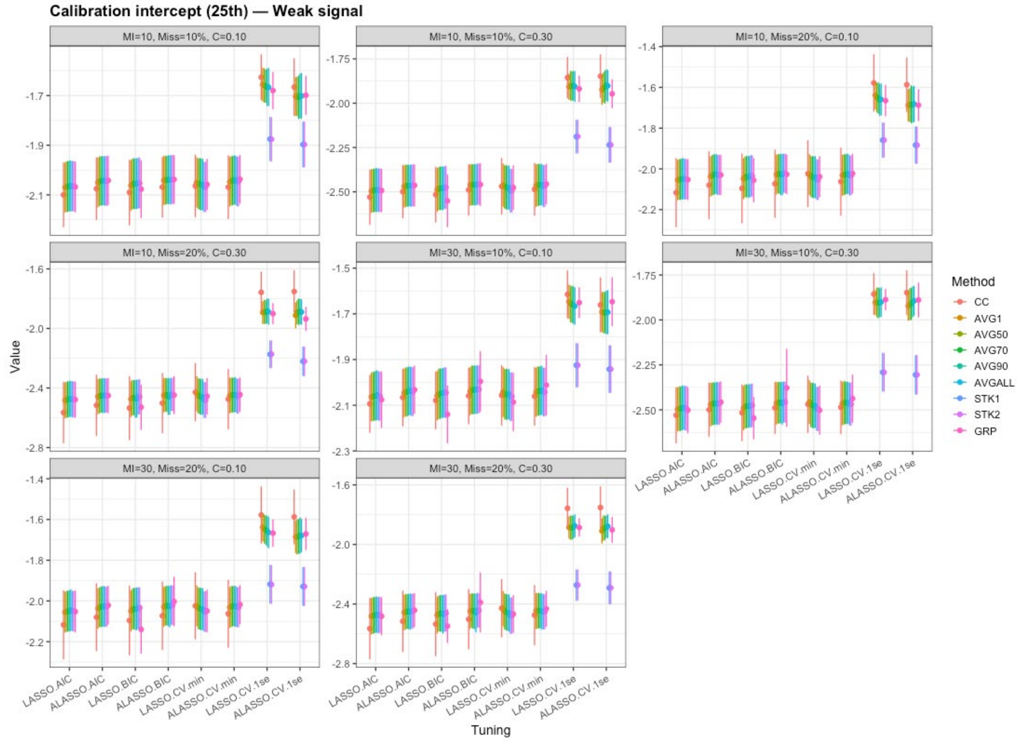

Supplemental Figure S10. Calibration intercept at the 25th percentile (mean and SD) for **weak** signal scenarios across MI levels, missingness (10%, 20%), and censoring (0.10, 0.30).

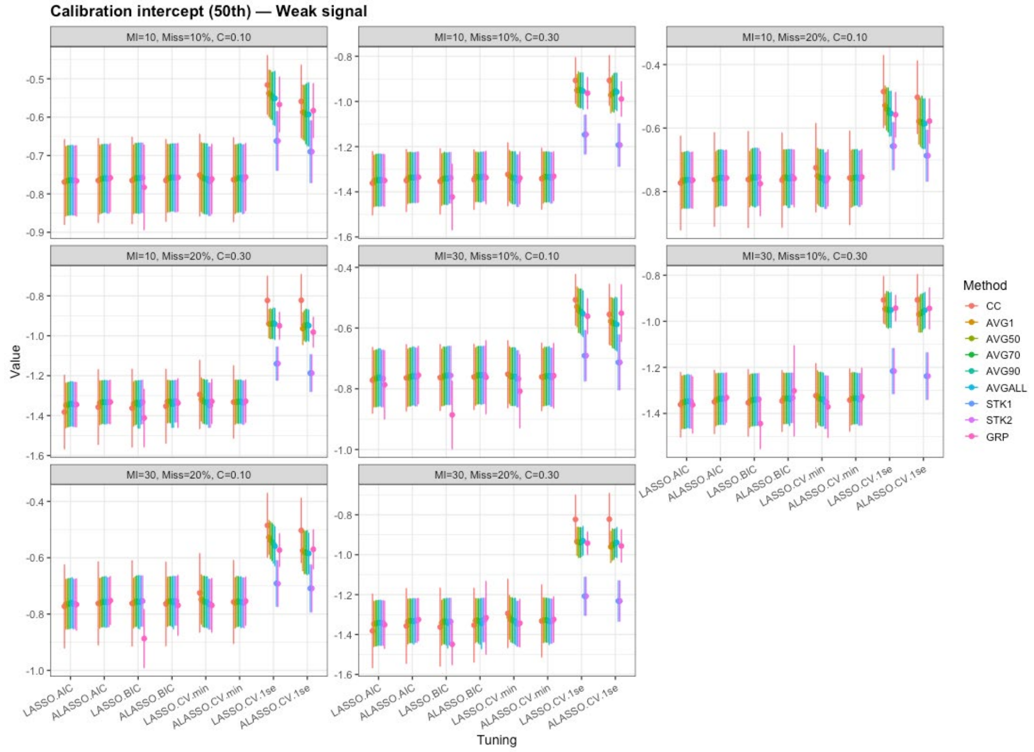

Supplemental Figure S11. Calibration intercept at the 50th percentile (mean and SD) for **weak** signal scenarios across MI levels, missingness (10%, 20%), and censoring (0.10, 0.30).

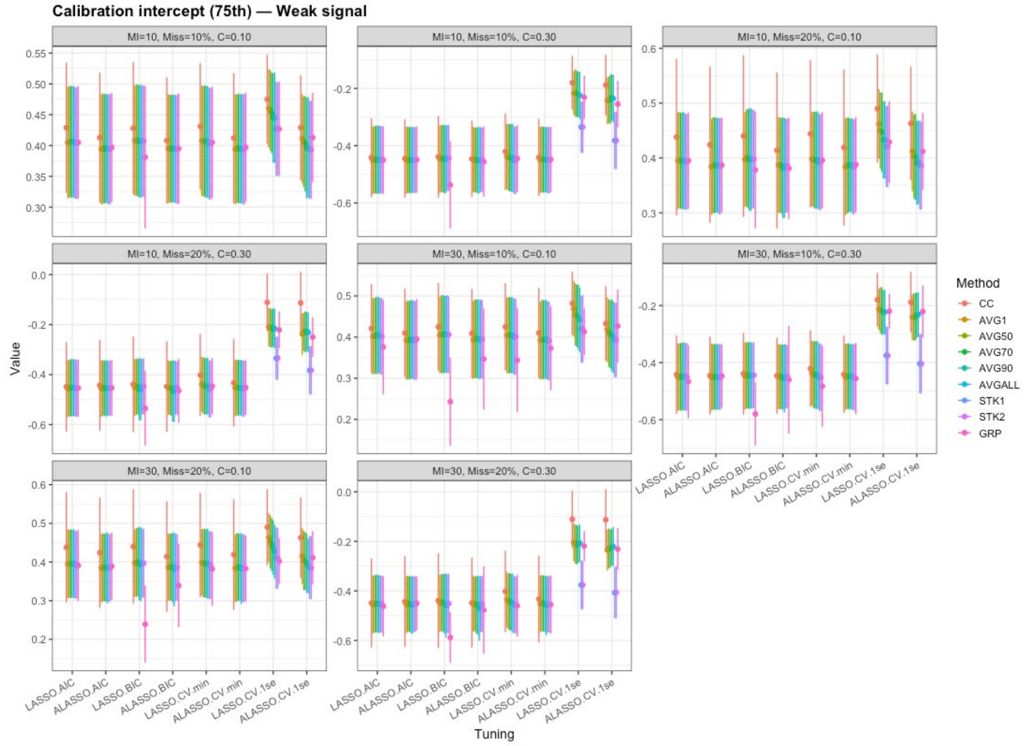

Supplemental Figure S12. Calibration intercept at the 75th percentile (mean and SD) for **weak** signal scenarios across MI levels, missingness (10%, 20%), and censoring (0.10, 0.30).

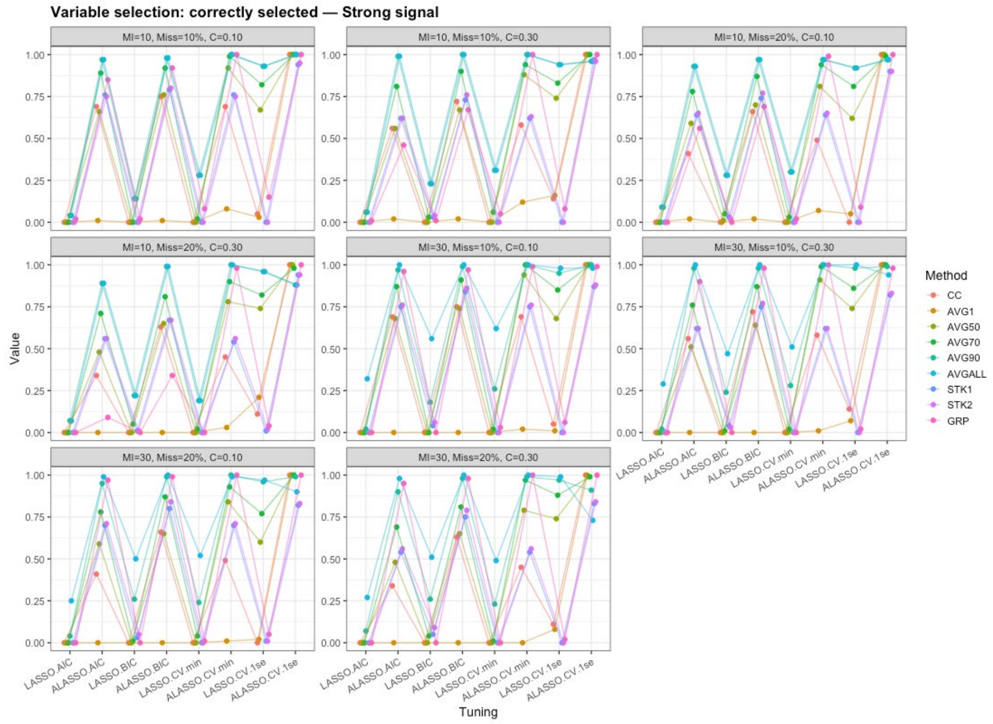

Supplemental Figure S13. Variable selection—correctly selected predictors for **strong** signal scenarios across MI levels, missingness (10%, 20%), and censoring (0.10, 0.30).

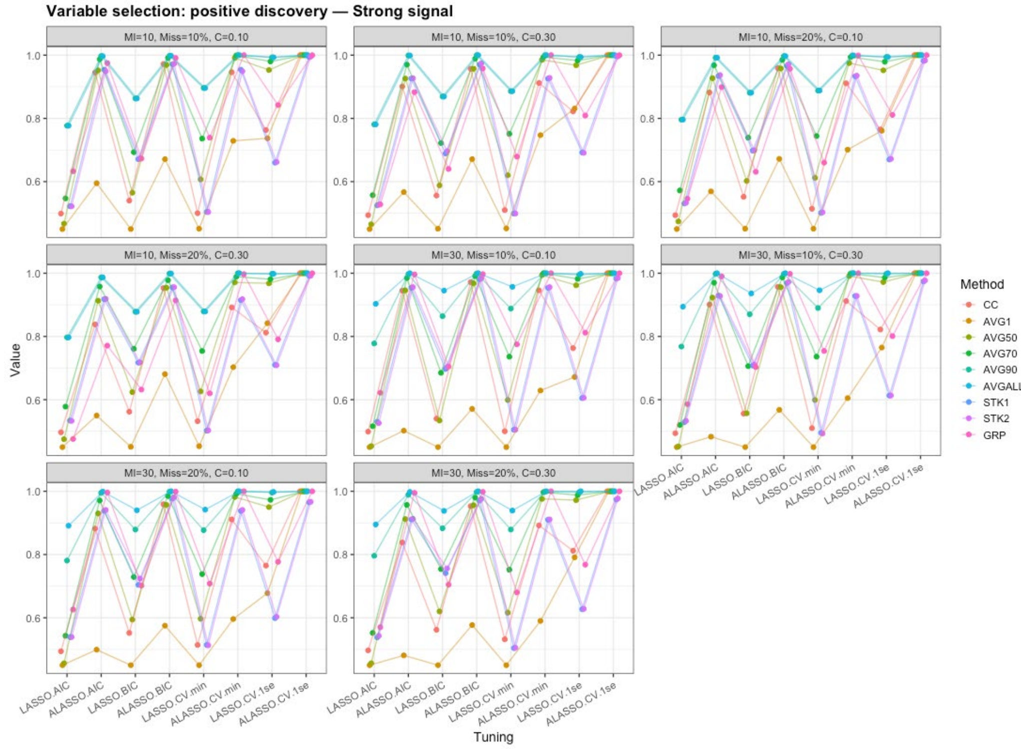

Supplemental Figure S14. Variable selection—positive discovery / selection rate for **strong** signal scenarios across MI levels, missingness (10%, 20%), and censoring (0.10, 0.30).

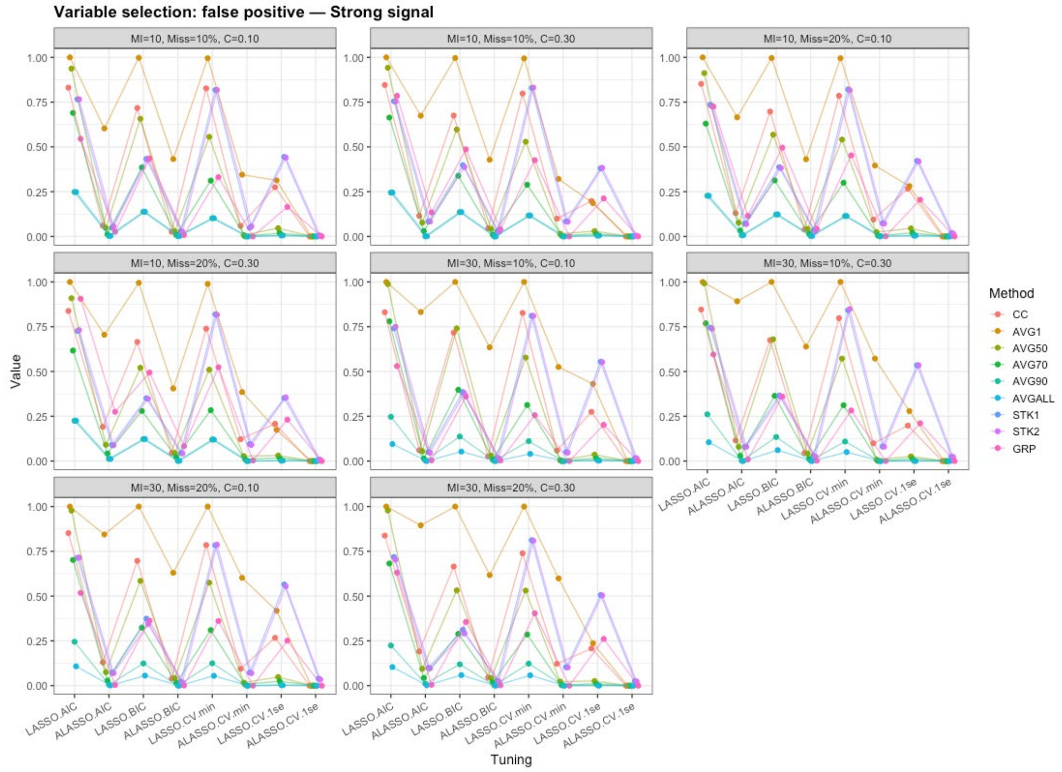

Supplemental Figure S15. Variable selection—false positives for **strong** signal scenarios across MI levels, missingness (10%, 20%), and censoring (0.10, 0.30).

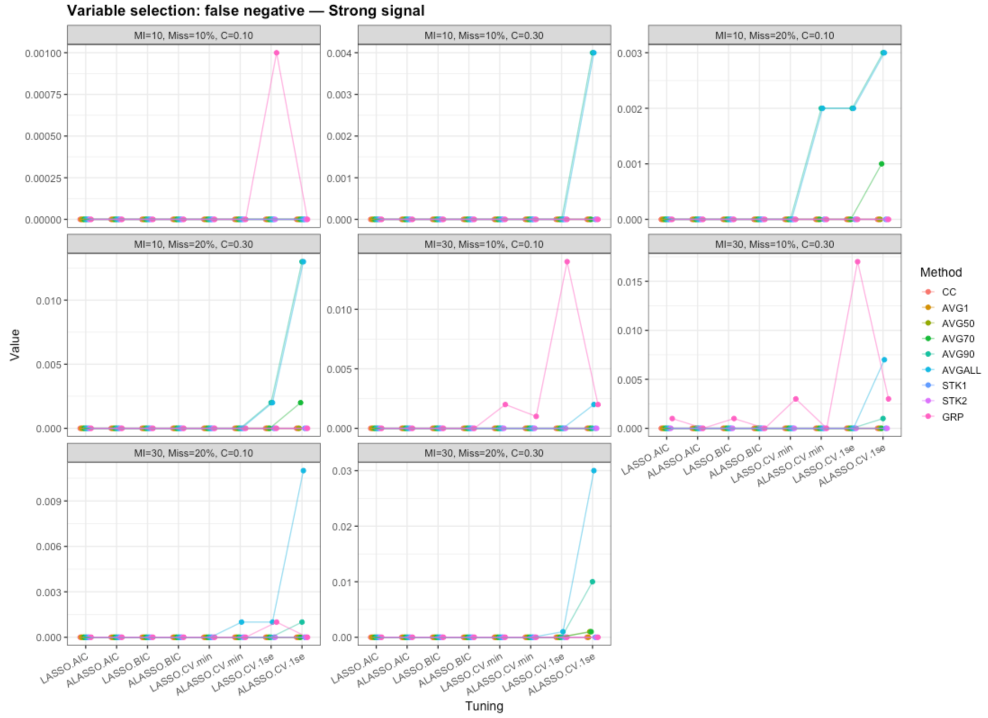

Supplemental Figure S16. Variable selection—false negatives for **strong** signal scenarios across MI levels, missingness (10%, 20%), and censoring (0.10, 0.30).

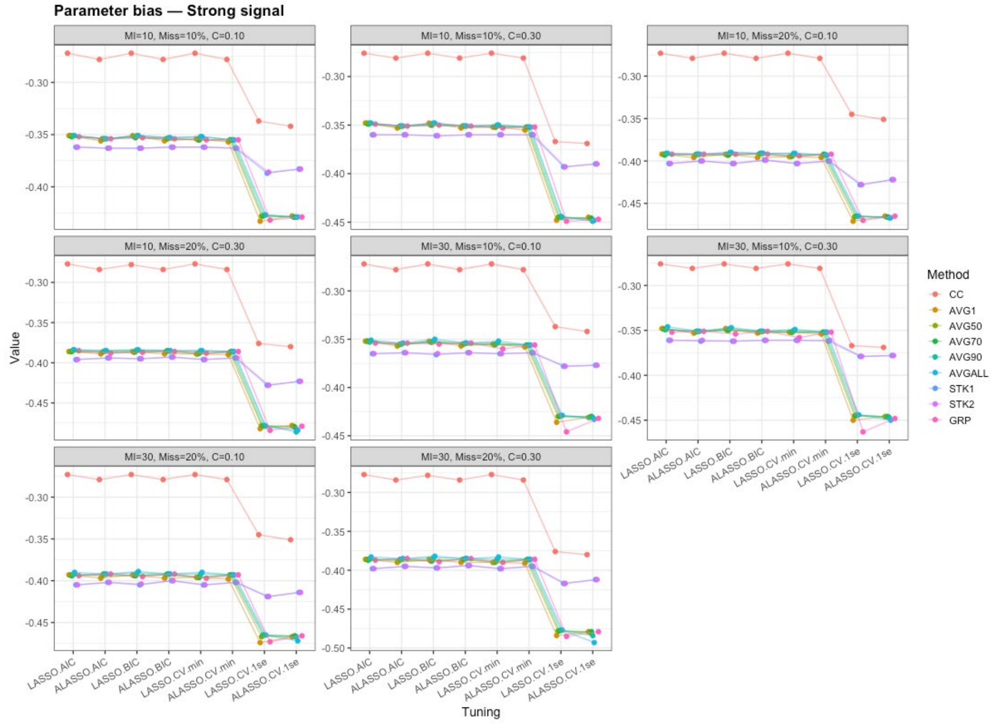

Supplemental Figure S17. Parameter estimation—average bias across methods and tuning settings for **strong** signal scenarios across MI levels, missingness (10%, 20%), and censoring (0.10, 0.30).

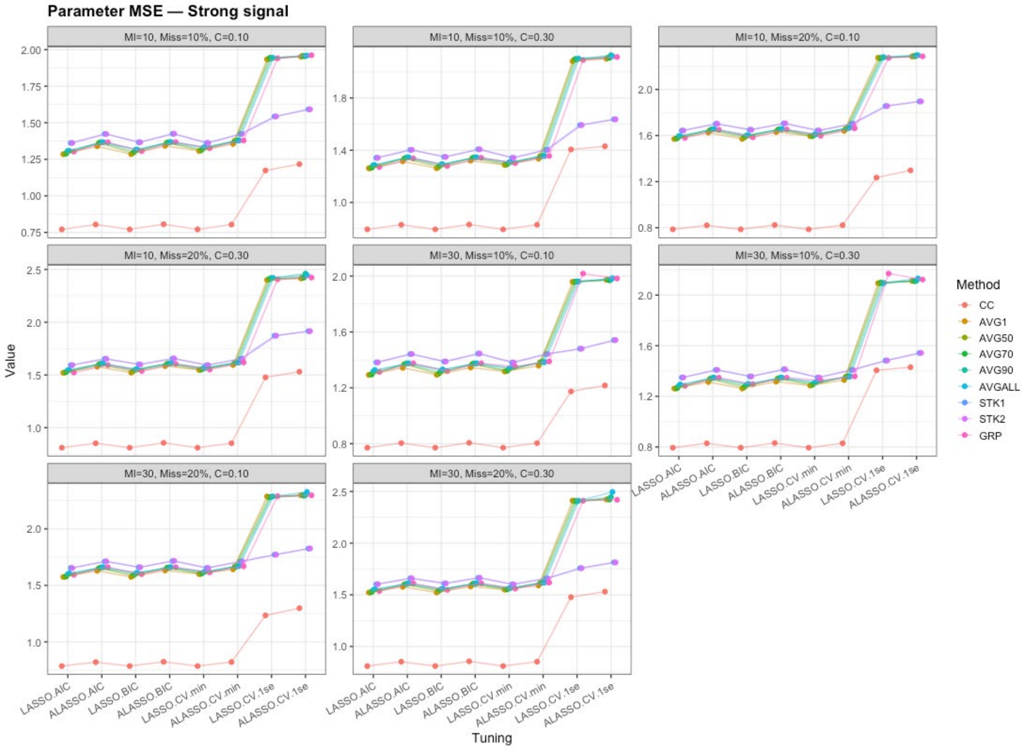

Supplemental Figure S18. Parameter estimation—average MSE across methods and tuning settings for **strong** signal scenarios across MI levels, missingness (10%, 20%), and censoring (0.10, 0.30).

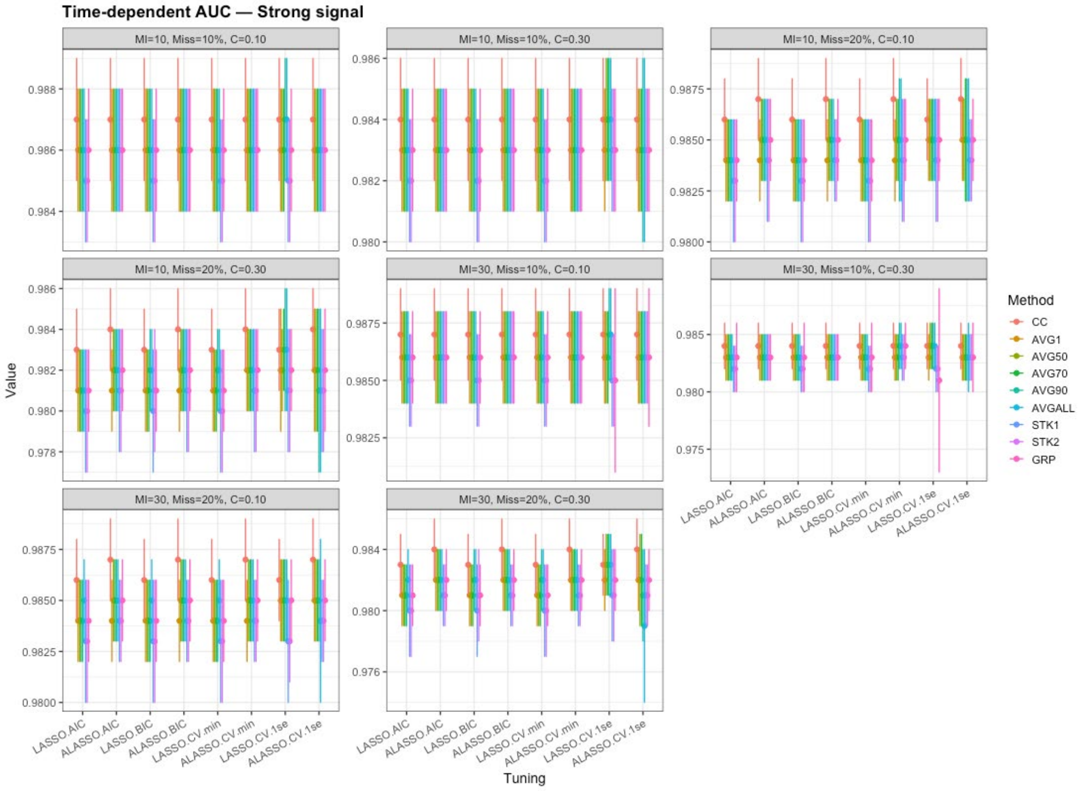

Supplemental Figure S19. tAUC (mean and SD) from ridge refits for **strong** signal scenarios across MI levels, missingness (10%, 20%), and censoring (0.10, 0.30).

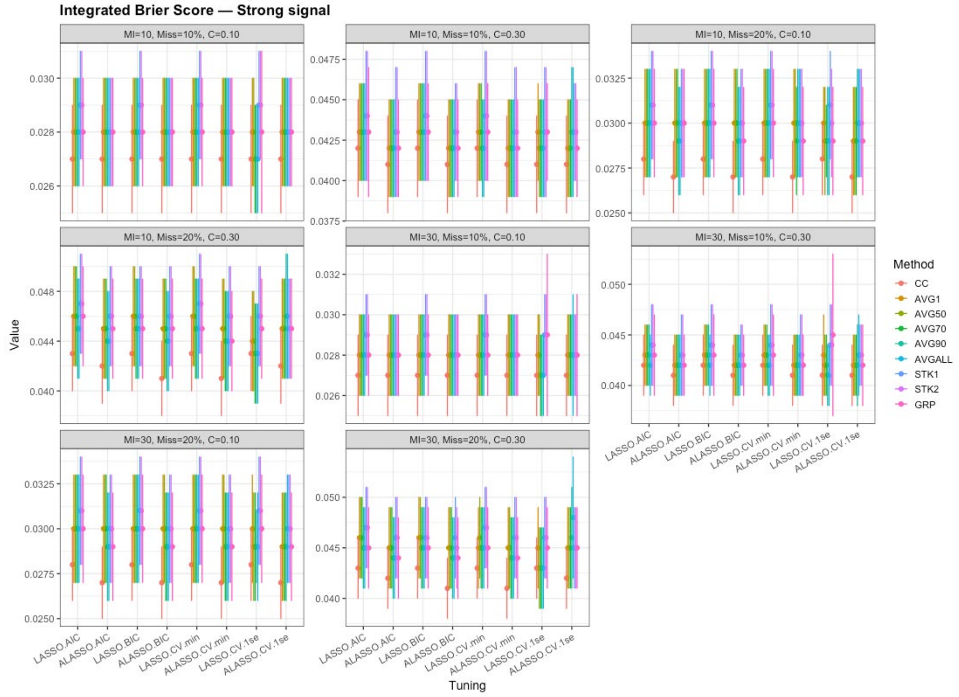

Supplemental Figure S20. Integrated Brier Score (mean and SD) for **strong** signal scenarios across MI levels, missingness (10%, 20%), and censoring (0.10, 0.30).

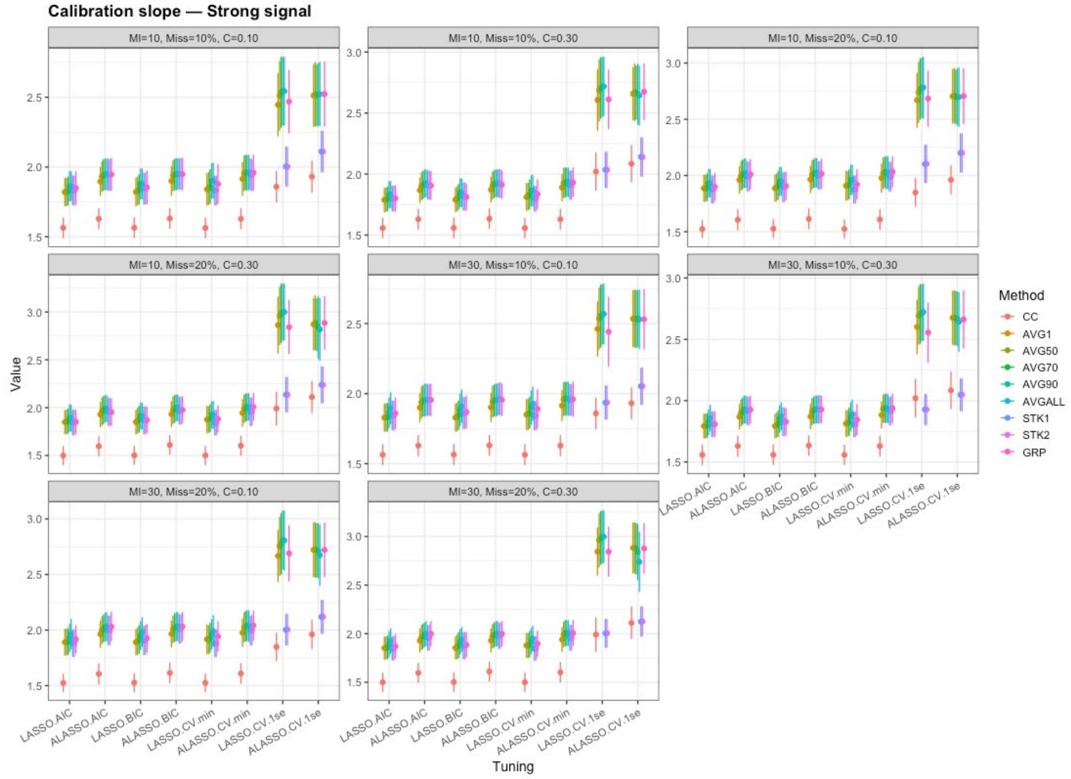

Supplemental Figure S21. Calibration slope (mean and SD) for **strong** signal scenarios across MI levels, missingness (10%, 20%), and censoring (0.10, 0.30).

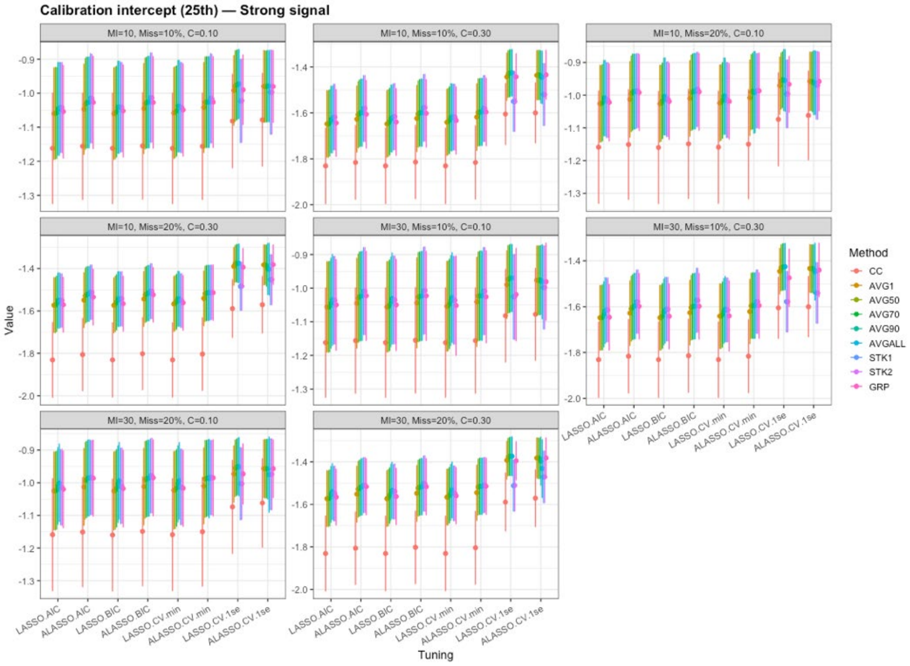

Supplemental Figure S22. Calibration intercept at the 25th percentile (mean and SD) for **strong** signal scenarios across MI levels, missingness (10%, 20%), and censoring (0.10, 0.30).

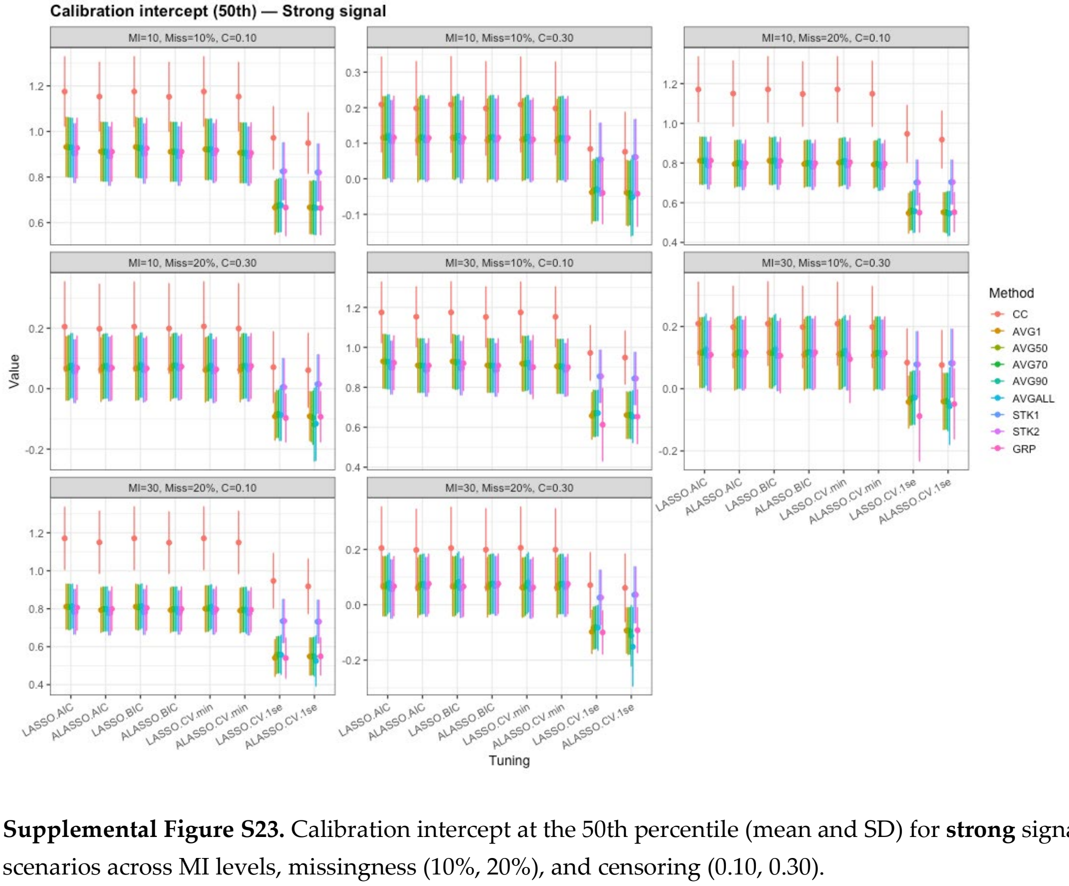

Supplemental Figure S23. Calibration intercept at the 50th percentile (mean and SD) for **strong** signal scenarios across MI levels, missingness (10%, 20%), and censoring (0.10, 0.30).

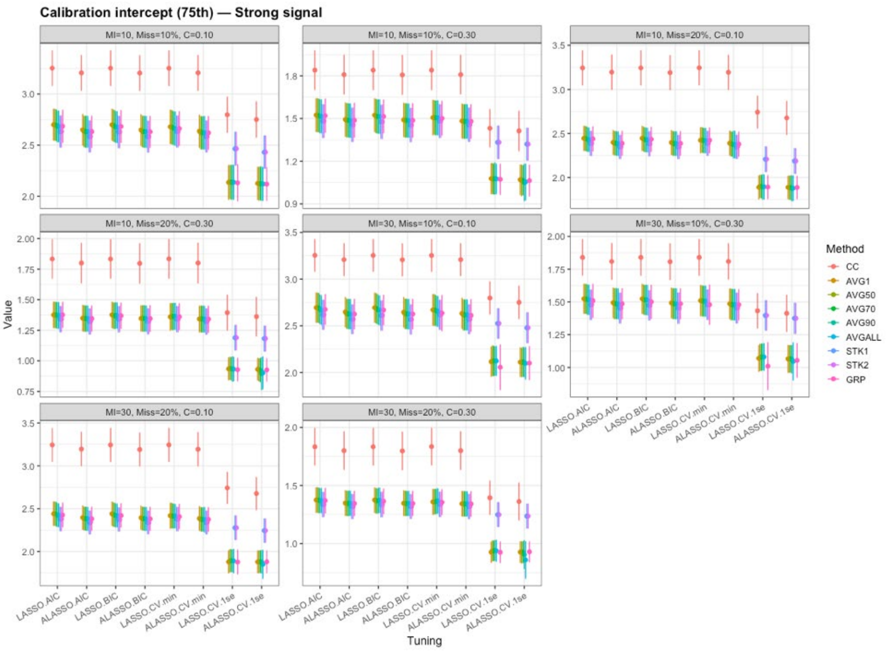

Supplemental Figure S24. Calibration intercept at the 75th percentile (mean and SD) for **strong** signal scenarios across MI levels, missingness (10%, 20%), and censoring (0.10, 0.30).

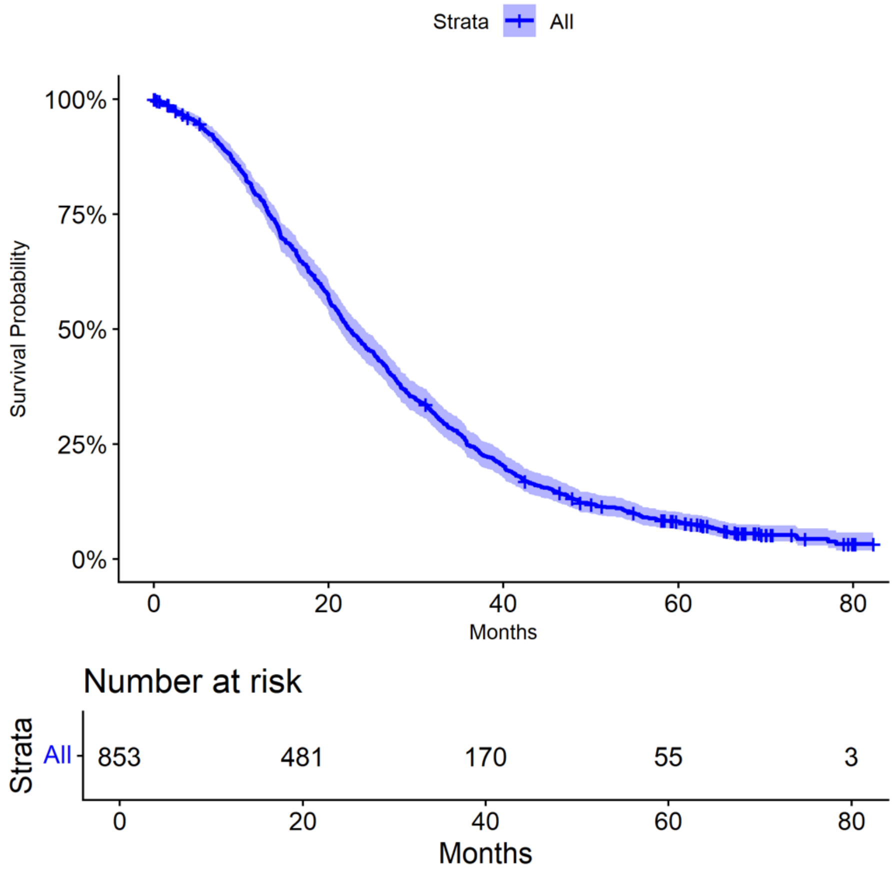

Supplemental Figure S25. Kaplan–Meier curve of overall survial in the CALGB 90401 data.
